# Epithelial tension in the second heart field promotes mouse heart tube elongation

**DOI:** 10.1038/ncomms14770

**Published:** 2017-03-30

**Authors:** Alexandre Francou, Christopher De Bono, Robert G. Kelly

**Affiliations:** 1 Aix-Marseille University, CNRS UMR 7288, Developmental Biology Institute of Marseille, Campus De Luminy Case 907, 13288 Marseille Cedex 9, France

## Abstract

Extension of the vertebrate heart tube is driven by progressive addition of second heart field (SHF) progenitor cells to the poles of the heart. Defects in this process cause a spectrum of congenital anomalies. SHF cells form an epithelial layer in splanchnic mesoderm in the dorsal wall of the pericardial cavity. Here we report oriented cell elongation, polarized actomyosin distribution and nuclear YAP/TAZ in a proliferative centre in the posterior dorsal pericardial wall during heart tube extension. These parameters are indicative of mechanical stress, further supported by analysis of cell shape changes in wound assays. Time course and mutant analysis identifies SHF deployment as a source of epithelial tension. Moreover, cell division and oriented growth in the dorsal pericardial wall align with the axis of cell elongation, suggesting that epithelial tension in turn contributes to heart tube extension. Our results implicate tissue-level forces in the regulation of heart tube extension.

## Abstract (web summary)

Epithelial progenitor cell growth in the second heart field contributes to heart morphogenesis but how this is regulated at the tissue level is unclear. Here, the authors show that cell elongation, polarized actomyosin and nuclear YAP/TAZ drive epithelial growth and correlate with mechanical tension.

Epithelial remodelling during embryonic development is a critical process in establishing body shape and organogenesis and is driven by a complex combination of cell and tissue-level forces^1^,^2^,^3^. The heart tube in the early vertebrate embryo is derived from epithelial cardiac progenitor cells in splanchnic mesoderm^4^,^5^. The heart subsequently elongates and loops as second heart field (SHF) progenitor cells in the dorsal wall of the pericardial cavity (DPW) contribute to the growing arterial and venous poles^6^,^7^. Defects in SHF deployment cause a spectrum of common congenital heart defects^7^,^8^. SHF cells in the DPW form an epithelial layer contiguous with the cardiac poles during heart tube elongation (embryonic day (E) 8.5–10.5) (refs 9, 10, 11, 12, 13). Clonal analysis, cell-tracing and genetic lineage experiments have shown that progenitor cells giving rise to arterial and venous pole myocardium segregate from a common progenitor pool in the posterior region of the SHF^14^,^15^,^16^,^17^. Recent studies have shown that apicobasal polarity regulates proliferation and differentiation in the SHF. In particular, cell shape changes in the SHF of mouse embryos lacking the 22q11.2 deletion syndrome candidate gene *Tbx1* are associated with loss of basal filopodia and elevated aPKCz levels contributing to decreased proliferation and ectopic differentiation in the *Tbx1*^*−/−*^ DPW^12^. Loss of N-cadherin in the SHF also perturbs the progenitor cell niche, resulting in defective progenitor cell renewal in the DPW^18^. The planar cell polarity gene *Vangl2* regulates epithelial organization in SHF cells as they differentiate into outflow tract (OFT) myocardium at the arterial pole of the heart, and loss of *Vangl2* leads to OFT septation defects^19^. Furthermore, increased epithelial cell cohesion in the anterior DPW (aDPW) has recently been proposed to create a pulling force that drives progenitor cell addition to the OFT^20^. Altogether, these studies identify the epithelial properties of cells in the DPW as a regulatory step in the control of proliferation, differentiation and deployment of cardiac progenitor cells.

Here we show that SHF cells in the DPW are subject to anisotropic mechanical stress, indicated by oriented cell elongation and deformation on wounding. The posterior DPW (pDPW) is characterized by elevated cell deformation, polarized actomyosin distribution and nuclear YAP/TAZ accumulation. These parameters are consistent with polarized epithelial tension in the DPW. Investigation of different stages of heart tube development, and mutant embryos in which heart tube elongation is perturbed, implicates SHF deployment as a source of mechanical force leading to epithelial tension. Furthermore, cell division and patterns of growth in the DPW are polarized along the axis of cell elongation, suggesting that epithelial stress in turn contributes to growth of the heart tube.

## Results

### Oriented cell elongation and mechanical stress in the DPW

**Figure 1:**
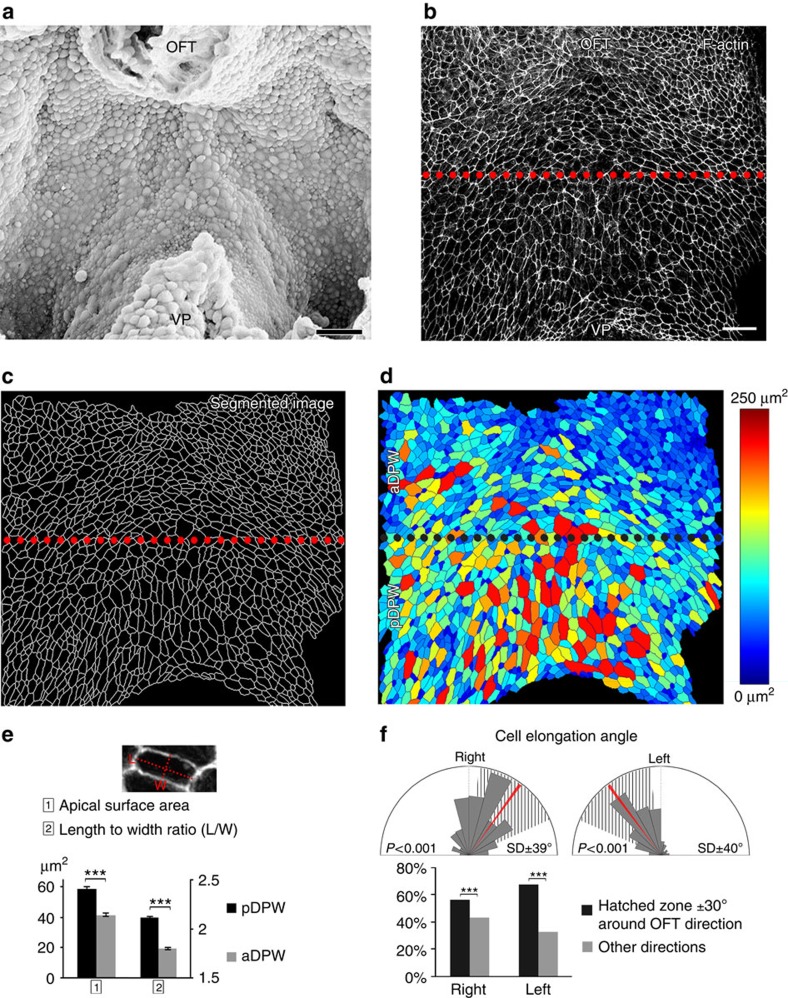
Oriented cell elongation in the DPW. (**a**) Ventral scanning electron micrograph of the DPW epithelium of an E9.5 embryo after removing the heart. Apical surface view of the DPW at E9.5 showing F-actin (**b**), apical membrane skeleton after segmentation (**c**) and a heat map of apical surface area (**d**). (**e**) Cells in the pDPW have a larger apical surface area and are more elongated than in the aDPW (*n*=3 embryos, pDPW: 1,522 cells, aDPW: 1,688 cells). (**f**) Cells are elongated on an axis oriented towards the arterial pole (red bar) (*n*=3 embryos, right: 1,462 cells, left: 1,671 cells; *P*-value based on a Rayleigh circular test). OFT: outflow tract, VP: venous pole. ***P<0.001 (**e**: Unpaired bilateral *t*-test; **f**: Chi-squared test). Error bars represent s.e.m. Scale bars, 30 μm.

We examined cell shape and organization in the plane of the DPW epithelium in ventral whole mount views of mouse embryos with the heart removed at embryonic day (E) 9.5 (Fig. 1a). Apical cell membranes were identified by Phalloidin staining of cortical F-Actin and the DPW was imaged from the apical surface using confocal microscopy (Fig. 1b). Segmentation software was used to isolate and identify individual cells throughout the DPW and quantify cellular parameters (Fig. 1c,d, Supplementary Fig. 1)^21^. This analysis revealed that cells in the DPW have an elongated shape and that cells in the pDPW (pDPW, here defined as the posterior half of the epithelium, Fig. 1b), close to the venous pole of the heart, have a larger apical surface area and are more elongated than cells in the aDPW (aDPW, anterior half of the epithelium), close to the arterial pole (Fig. 1e). Measurement of the direction of cell elongation identified an elongation axis directed towards the arterial pole of the heart (Fig. 1f, Supplementary Fig. 1).

**Figure 2:**
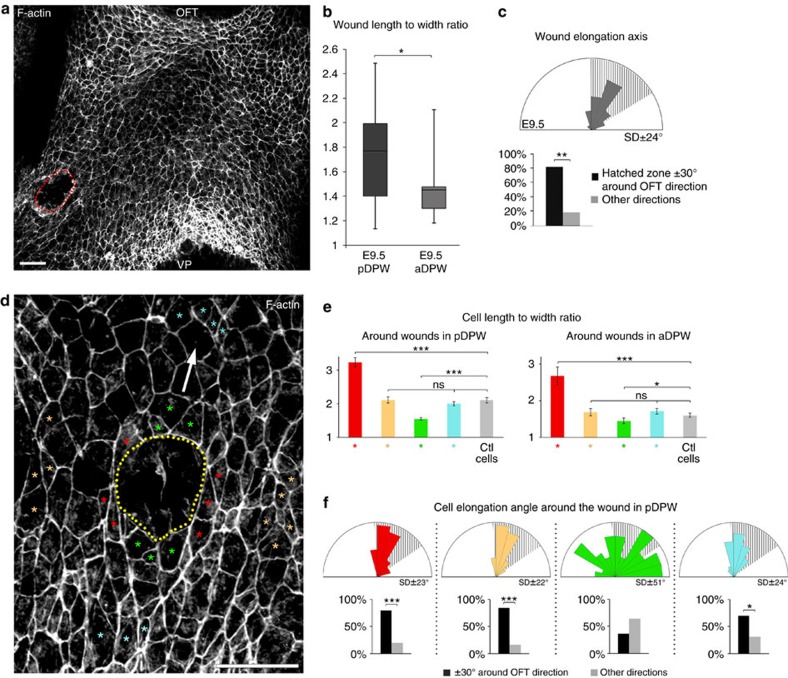
Oriented epithelial stress in the DPW epithelium. (**a**) Circular wounds were made on the right side of the DPW epithelium of living embryos using drawn glass pipettes. (**b**) Analysis of embryos fixed 2–5 min after wounding showed that wounds are highly elongated in the pDPW of E9.5 embryos and less so in the aDPW (determined by the length to width ratio) (pDPW: *n*=22 embryos, aDPW: *n*=10 embryos). In the box plot, the centre lines show the median, box limits show the first and third quartiles, and whiskers show maximum and minimum values. (**c**) Wounds generated in the aDPW and pDPW of E9.5 embryos are significantly elongated on an axis directed towards the OFT. (**d**) F-actin staining showing a wound in the DPW (dotted line); cells around the wound have altered shapes depending on their position relative to the wound. Coloured asterisks identify representative cells measured in **e**,**f** and the arrow indicates the direction of the OFT. (**e**) Length-to-width ratio in cells anterior and posterior to the wound (green), lateral to the wound (red), 3–4 cell rows away from the wound (yellow and light blue) and contralateral control cells (grey). Cell elongation is altered in cells around the wound (pDPW: *n*=11 embryos, 65 red cells, 57 yellow cells, 61 green cells, 59 light blue cells and 69 control cells; aDPW: *n*=4 embryos, 18 red cells, 32 yellow cells, 21 green cells, 20 light blue cells and 29 control cells). (**f**) Cell elongation axis in the wound region in the pDPW showing altered elongation axes of cells anterior and posterior to the wound (green). OFT: outflow tract, VP: venous pole. *P<0.05; **P<0.01; ***P<0.001 (**b**,**e**: Unpaired bilateral Mann–Whitney test; **c**,**f**: Chi-squared test). Error bars represent s.e.m. Scale bars, 30 μm.

Oriented cell elongation in epithelia can result from mechanical forces creating anisotropic tension and cell stretching during tissue morphogenesis^1^,^2^,^3^. We used a wound assay in order to investigate mechanical stress in the DPW epithelium. A circular wound, generated using a drawn glass pipette, was made in the pDPW or aDPW of living embryos. Analysis after 2–5 min revealed that the majority of wounds became elongated on an axis oriented towards the arterial pole (Fig. 2a–c, Supplementary Fig. 2). The extent of elongation was greater in the pDPW compared with the aDPW (Fig. 2b). Measurement of the shape of cells directly surrounding wounds in both the pDPW and aDPW revealed that cells lateral to the wound were significantly more elongated compared with contralateral control cells (Fig. 2d,e, red cells and Supplementary Figs 2 and 3), whereas cells anterior and posterior to the wound were less elongated (Fig. 2d,e green cells and Supplementary Figs 2 and 3), suggesting an altered distribution of epithelial stress around the wound. Cell shape changes measured in the aDPW were less than in the pDPW (Fig. 2e). The shape of cells 3–4 cell rows away from the wound was not significantly different from that of contralateral control cells (Fig. 2d,e yellow and light blue cells, and Supplementary Figs 2 and 3). In addition to shape changes, cells anterior and posterior to the wound displayed altered orientation compared with control cells, whereas the direction of elongated cells on the sides of the wound was maintained (Fig. 2f and Supplementary Figs 2 and 3). These results are consistent with cells in the DPW being subject to polarized mechanical stress that deforms cells on an axis directed towards the arterial pole; furthermore, wounding locally changes the pattern of cell deformation in the tissue.

### Polarized actomyosin in the pDPW limits cell deformation

**Figure 3:**
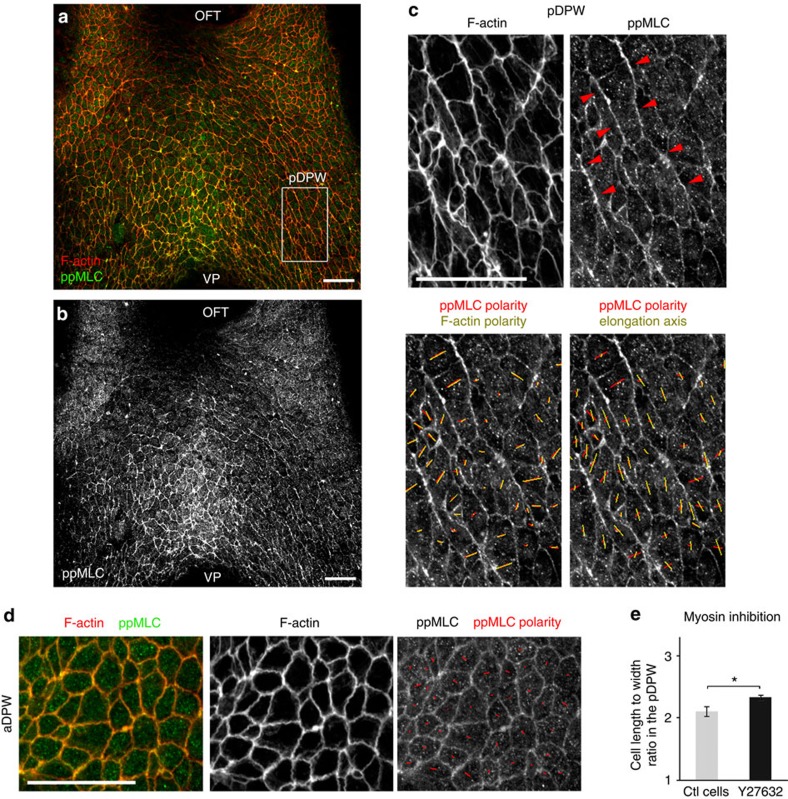
Polarized actomyosin distribution in the posterior DPW. (**a**,**b**) Diphosphorylated myosin light chain (ppMLC) accumulates in the pDPW and is polarized on the long membranes of cells. (**c**) High magnification of the pDPW (box in **a**), showing polarized F-actin and ppMLC2 on long apical membranes and multicellular actomyosin cables (red arrowheads). (**d**) High magnification of the aDPW, showing low-level ppMLC2 labelling, homogeneously distributed at the apical membrane. (**e**) Cell elongation in the pDPW increases after Myosin inhibition by the ROCK inhibitor Y27632 (*n*=7 embryos, right: 265 cells). Red and yellow bars in **c**,**d** indicate nematic polarity of ppMLC2, F-actin and cell elongation axis. OFT: outflow tract, VP: venous pole. *P<0.05 (Unpaired bilateral *t*-test). Error bars represent s.e.m. Scale bars, 30 μm.

Elongated epithelial cells in *Drosophila* and in chick rearrange their actomyosin cytoskeleton during tissue morphogenesis, and actomyosin activity can drive and/or respond to membrane movement and cell deformation^22^,^23^,^24^. We analysed the distribution of active myosin complexes and found that diphosphorylated non-muscle myosin light chain 2 (ppMLC2) accumulates predominantly in the pDPW with a strong signal in the medial region close to the venous pole (Fig. 3a–d). Strikingly, both ppMLC2 and F-actin are enriched on the long membranes of elongated cells (Fig. 3c). We observed multicellular actomyosin cables along the long membranes of adjacent cells (2–10 cell lengths), oriented parallel to the axis of individual cell elongation (Fig. 3c). In contrast, low-level homogeneous ppMLC2 distribution was observed around less elongated cells in the aDPW (Fig. 3d). Polarized cables on the long membranes of elongated cells in the pDPW were also observed with an antibody detecting monophosphorylated myosin light chain (pMLC) (Supplementary Fig. 4). We examined the effect of blocking myosin activity on cell shape in the DPW using a ROCK inhibitor in embryo culture (Supplementary Fig. 4). Quantification of cell shape in the pDPW revealed a small but significant increase of cell elongation in cells from treated versus control embryos after 40 min of culture (Fig. 3e). Actomyosin activity thus reduces tissue deformation in the DPW by counteracting cell elongation. Together these results suggest that polarized actomyosin complexes are a response to oriented mechanical forces in the DPW epithelium and limit cell deformation.

### Oriented mechanical stress in the DPW during SHF deployment

**Figure 4:**
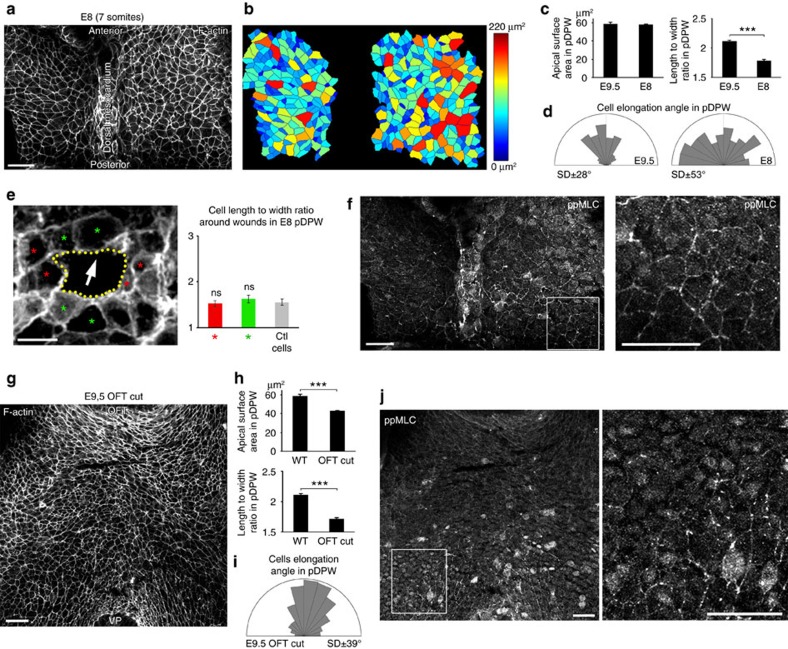
Epithelial tension in the DPW during heart tube elongation. (**a**) Apical view at E8 (seven somites) and (**b**) a heat map of apical surface area showing homogeneous cell size along the anterior-posterior axis of the DPW. (**c**) pDPW cells at E8 have a similar apical surface area compared with E9.5 but are less elongated and randomly oriented (**d**) (E9.5 *n*=3 embryos, 1,522 cells; E8 *n*=5 embryos, 468 cells). (**e**) Wound assay in the pDPW of E8 embryos (the arrow indicates the direction of the arterial pole). Cells anterior and posterior (green asterisk), and lateral to the wound (red asterisk) do not have altered length-to-width ratios (reflecting cell elongation) compared with control cells (*n*=5 embryos, 22 red cells, 22 green cells and 33 control cells). (**f**) At E8 ppMLC2 is homogenous through the DPW and not polarized (high-magnification box), although multicellular cables are observed in the dorsal mesocardium. (**g**) Apical view after heart tube transection and six hours of embryo culture. (**h**) pDPW cells have a smaller apical surface and are less elongated than in control embryos but remain oriented (**i**) (*n*=8 embryos, 637 cells). (**j**) ppMLC2 staining is reduced and multicellular cables are not apparent (high-magnification box) after OFT transection. OFT: outflow tract, VP: venous pole. ***P<0.001 (Unpaired bilateral *t*-test). Error bars represent s.e.m. Scale bars, **a**,**f**,**g**,**j**, 30 μm; **e**, 10 μm.

We next investigated the hypothesis that oriented mechanical stress in the DPW results from forces acting across the epithelium as SHF cells contribute to the cardiac poles. To test this we investigated cell shape, mechanical stress and actomyosin distribution in the DPW at different stages of heart tube formation as well as in experimentally and genetically manipulated embryos. At E8 the heart tube is connected to the DPW epithelium along its entire length by the dorsal mesocardium. Quantification revealed that cell apical surface area is homogeneous throughout the DPW at this stage and similar to that of cells in the pDPW at E9.5, although cells are less elongated and not directionally oriented (Fig. 4a–d). We performed a wound assay in the pDPW of E8 embryos. Wounds were significantly less elongated than at E9.5 and not specifically oriented towards the arterial pole (Fig. 4e and Supplementary Figs 2 and 3). Moreover, analysing the shape of the cells around the wounds revealed that cell length and orientation were indistinguishable from those of contralateral control cells (Fig. 4e and Supplementary Fig. 3). ppMLC2 was homogeneously distributed in the DPW at E8 and no multicellular cables were observed, with the exception of the region of the dorsal mesocardium (Fig. 4f). Together these results suggest that cells in the E8 DPW epithelium are less deformed and less subject to polarized epithelial forces compared with the situation at E9.5. At E8.5 the dorsal mesocardium breaks down, restricting addition of SHF cells to the cardiac poles. After this timepoint elongated cells, polarized ppMLC2 accumulation and oriented myosin cables are observed in the DPW (Supplementary Fig. 5). When SHF addition is complete, at E10.75, only low levels of ppMLC2 are detected in the residual DPW (Supplementary Fig. 5). Epithelial cell elongation, oriented mechanical force and polarized distribution of actomyosin in the DPW thus temporally correlate with SHF deployment to the cardiac poles.

To further investigate the origin of mechanical stress in the DPW, we transected the OFT before embryo culture. Cells in the pDPW have a smaller apical surface area and are less elongated in experimental than control embryos (Fig. 4g,h), although the axis of elongation remains oriented towards the arterial pole (Fig. 4i). Furthermore, only residual ppMLC2 labelling was observed in the posterior region of embryos 6 h after transection (Fig. 4j). This result suggests that cell elongation and the polarized distribution of actomyosin in the DPW requires continuity with an intact heart tube.

**Figure 5:**
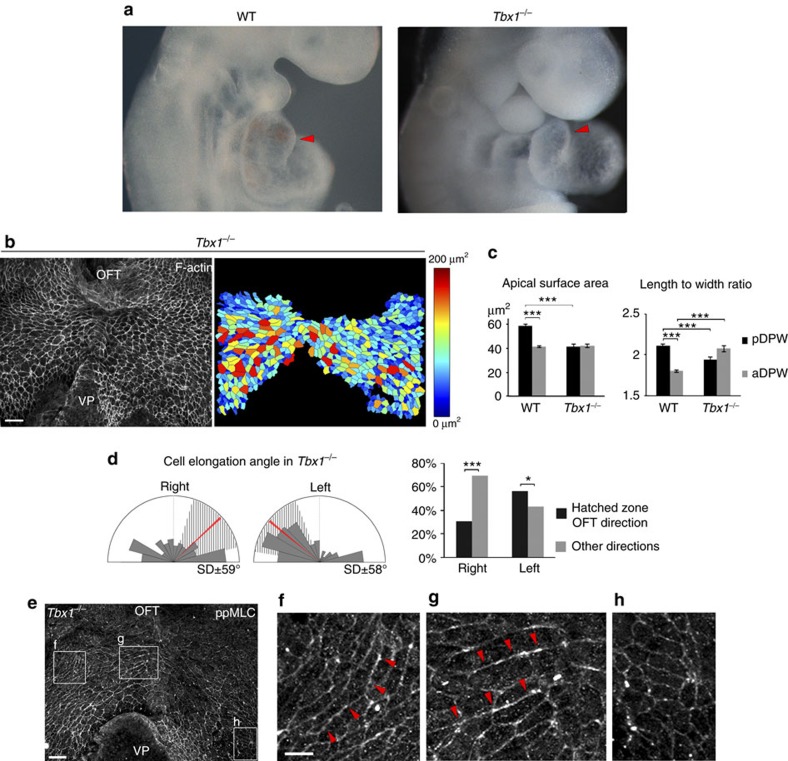
Altered epithelial stress in the DPW of *Tbx1* mutant embryos. (**a**) *Tbx1*^*−/−*^ embryos have a shorter OFT than wild-type embryos (red arrowheads). (**b**) DPW of an E9.5 *Tbx1*^*−/−*^ embryo and heat map of apical surface area. (**c**) Apical surface area and cell elongation abnormalities in *Tbx1*^*−/−*^ embryos (*n*=3 embryos, pDPW: 657 cells, aDPW: 455 cells). (**d**) Diagram showing that cell elongation in *Tbx1*^*−/−*^ embryos is not restricted to an axis directed towards the arterial pole (*n*=3 embryos, right: 388 cells, left 516 cells). (**e**) ppMLC2 staining showing abnormal distribution of actomyosin cables in a *Tbx1*^*−/−*^ embryo (**f**,**g**) and a low level of non-polarized myosin in the pDPW (**h**). *P<0.05; ***P<0.001 (**c**: Unpaired bilateral .*t*-test; **d**: Chi-squared test). Error bars represent s.e.m. Scale bars, **b**,**e**: 30 μm; **f** high mag, 10 μm.

**Figure 6:**
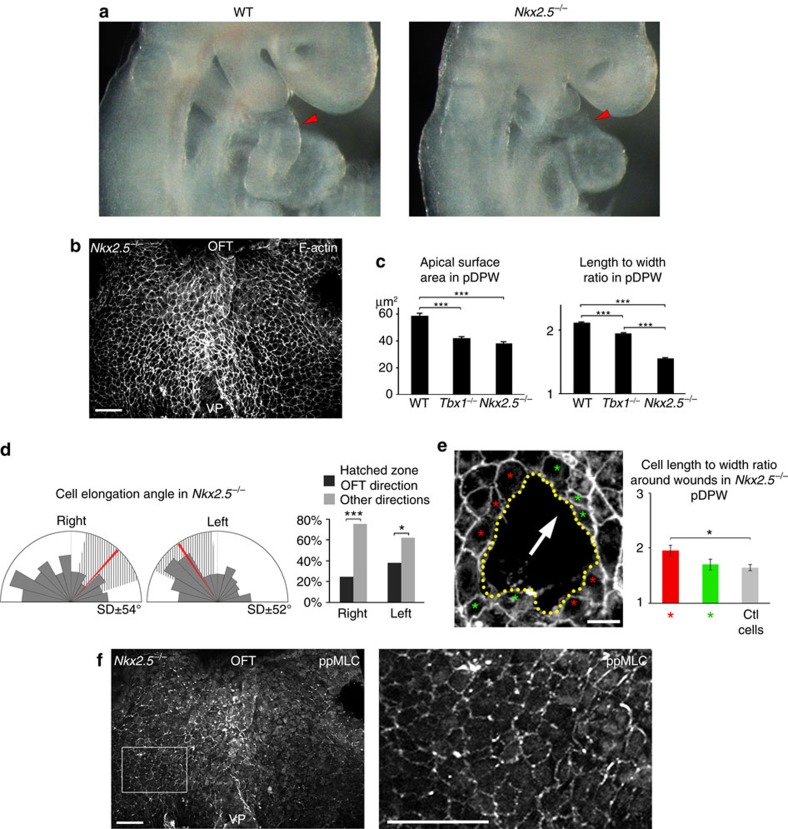
Reduced epithelial stress in the DPW of *Nkx2-5* mutant embryos. (**a**) *Nkx2-5*^*−/−*^ embryos have a severe defect in heart tube elongation compared with wild-type embryos (red arrowheads). (**b**) DPW of an E9.5 *Nkx2-5*^*−/−*^ embryo after phalloidin staining. (**c**) Apical surface area and elongation of pDPW cells in *Nkx2-5*^*−/−*^ embryos are reduced compared with cells in wild-type embryos (WT *n*=3 embryos, pDPW: 1,522 cells, aDPW: 1,688 cells; *Nkx2-5*^*−/−*^ pDPW *n*=6 embryos, 356 cells). (**d**) Elongation of cells in the pDPW of *Nkx2-5*^*−/−*^ embryos cells is not restricted to an axis directed towards the arterial pole (*n*=6 embryos, right: 201 cells, left: 155 cells). (**e**) Wound assay in the pDPW of *Nkx2-5*^*−/−*^ embryos (the arrow indicates the direction of the arterial pole). Cell shape in cells anterior and posterior (green asterisk), and lateral to the wound (red asterisk) is less disrupted than in wild-type embryos (*n*=5 embryos, 25 red cells, 28 green cells and 43 control cells). (**f**) ppMLC accumulates at a low level in the DPW of *Nkx2-5*^*−/−*^ embryos and is neither polarized nor forms multicellular cables (high-magnification boxed). *P<0.05; ***P<0.001 (**c**: Unpaired bilateral *t*-test; **d**: Chi-squared test; **e**: Unpaired bilateral Mann–Whitney test). Error bars represent s.e.m. Scale bars, **b**,**f**: 30 μm; **e**, 10 μm.

We investigated cell shape, force patterns and actomyosin distribution in the DPW of mouse mutants with heart tube elongation defects^25^,^26^,^27^,^28^,^29^. In *Tbx1*^*−/−*^ embryos, the SHF is hypoplastic and progenitor cells in the DPW fail to deploy correctly and to maximally elongate the OFT^17^,^25^ (Fig. 5a). Epithelial cells in the DPW of *Tbx1*^*−/−*^ embryos are disorganized: pDPW cells have a smaller apical surface area and reduced elongation compared with wild type, whereas cell elongation is increased in the aDPW (Fig. 5b,c). Moreover, the direction of cell elongation throughout the DPW is highly perturbed, many cells being elongated on the embryonic left-right axis (Fig. 5b–d, Supplementary Fig. 1). The distribution of ppMLC2 is also disorganized in *Tbx1*^*−/−*^ embryos. Fewer cables could be observed in the pDPW and residual cables were found to display an abnormal orientation following that of *Tbx1*^*−/−*^ DPW cells (Fig. 5e–h). Epithelial disorganization is thus accompanied by abnormal cell deformation in the *Tbx1*^*−/−*^ DPW. In *Nkx2-5*^*−/−*^ embryos SHF deployment is perturbed and the heart tube fails to extend^29^ (Fig. 6a). We observed that cells in the *Nkx2-5*^*−/−*^ pDPW have smaller apical surface areas and are less elongated than in wild-type and *Tbx1*^*−/−*^ embryos (Fig. 6b,c). In addition, the direction of cell elongation is more random and significantly less oriented towards the arterial pole (Fig. 6d). Wound assays in the DPW of *Nkx2-5*^*−/−*^ embryos revealed that wounds were significantly less elongated than in wild-type embryos at E9.5 and less oriented towards the arterial pole (Fig. 6e and Supplementary Figs 2 and 3). Analysis of the shape of cells around the wounds revealed that cell elongation is less altered in *Nkx2-5*^*−/−*^ than in wild-type embryos with cell elongation axes oriented parallel to the wound border (Fig. 6e and Supplementary Fig. 2). These results are consistent with lower and more homogeneous patterns of epithelial stress in the DPW of *Nkx2-5*^*−/−*^ compared with wild-type embryos. In the absence of *Nkx2-5,* ppMLC2 is homogeneously distributed, not polarized at the cellular level and no actomyosin cables are observed, similar to the situation in the DPW of wild-type embryos at E8 (Fig. 6f). Nkx2-5 is expressed in the medial DPW of wild-type embryos, only partially overlapping with areas of elevated epithelial deformation in the pDPW (Supplementary Fig. 6). This suggests that altered epithelial stress in *Nkx2-5*^*−/−*^ embryos may be an indirect result of the requirement for Nkx2-5 in heart tube elongation. Together these results are consistent with a model by which oriented mechanical stress in the DPW is triggered by epithelial cell addition to the elongating heart tube.

### Active YAP in the pDPW promotes proliferation

**Figure 7:**
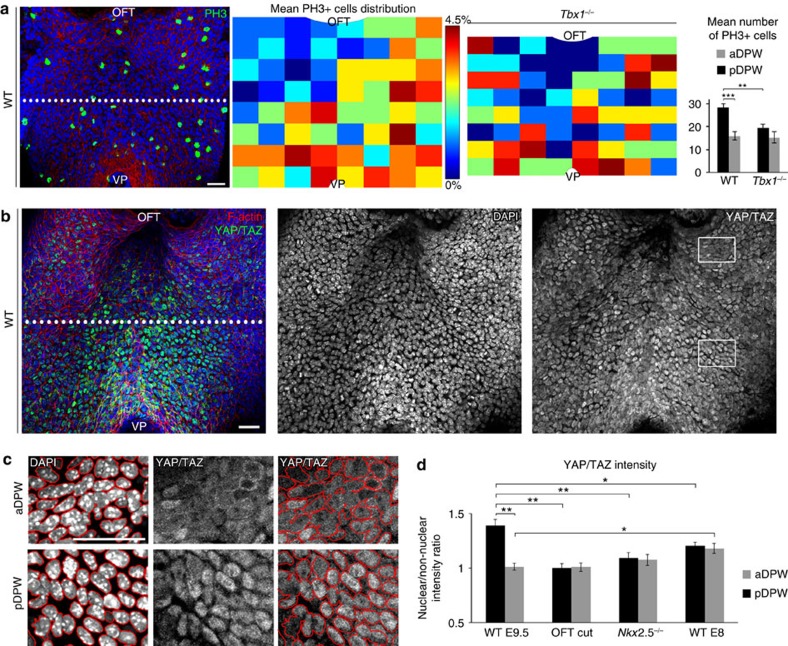
Proliferation and active YAP/TAZ in the pDPW. (**a**) Phospho-Histone H3 immunofluorescence staining in the DPW epithelium, heat maps of cumulative PH3+ cell distribution and plots showing a posterior growth centre in the pDPW of wild type but not *Tbx1*^*−/−*^ embryos (WT *n*=9 embryos; *Tbx1*^*−/−*^ *n*=4 embryos). (**b**) YAP/TAZ staining showing elevated nuclear labelling in the pDPW. (**c**) High-magnification views of aDPW and pDPW regions boxed in **b**. Nuclei were segmented and nuclear and non-nuclear YAP/TAZ intensity quantified. (**d**) Nuclear/non-nuclear YAP/TAZ intensity ratio showing significant nuclear YAP/TAZ accumulation in the pDPW of wild-type embryos but less nuclear accumulation after OFT transection, in *Nkx2-5*^*−/−*^ and in E8 wild-type embryos (WT E9.5 *n*=6 embryos; OFT cut *n*=8 embryos; *Nkx2-5*^*−/−*^ *n*=7 embryos; WT E8 *n*=5 embryos). *P<0.05; **P<0.01; ***P<0.001 (**a**: Unpaired bilateral *t*-test; **d**: Unpaired bilateral Mann–Whitney test). Error bars represent s.e.m. Scale bars, 30 μm.

Cell stretching and increased mechanical tension have been linked with elevated proliferation^2^,^30^,^31^. We quantified PH3-positive mitotic cells at E9.5 to generate a heat map of the distribution of proliferation in the murine DPW. Significantly more PH3-positive cells were observed in the pDPW compared with the aDPW, coinciding with the region of elevated epithelial tension at E9.5 and consistent with a study of proliferation in the avian SHF^32^ (Fig. 7a). In contrast, fewer PH3-positive cells were observed in the pDPW of *Tbx1*^*−/−*^ embryos (Fig. 7a and Supplementary Fig. 7) and throughout the DPW of *Nkx2-5*^*−/−*^ embryos (Supplementary Fig. 7), consistent with decreased proliferation in the SHF of *Tbx1*^*−/−*^ and *Nkx2-5*^*−/−*^ embryos^29^,^33^. We observed that PH3-positive cells were equivalently distributed in the pDPW and aDPW of E8 embryos (Supplementary Fig. 7). The posterior proliferative centre thus coincides temporally with the onset of cell elongation, oriented mechanical stress and polarized distribution of actomyosin, being observed only after breakdown of the dorsal mesocardium.

Nuclear localization of YAP/TAZ has been reported to mediate proliferation in response to mechanical cues in *Drosophila* and epithelial cells in culture^31^,^34^,^35^. Evaluation of YAP/TAZ expression revealed elevated nuclear (active) YAP/TAZ labelling in the pDPW where ppMLC2 accumulates; a strong signal was observed in the medial region close to the venous pole (Fig. 7b). Quantification of nuclear and non-nuclear YAP/TAZ intensity in the aDPW and pDPW revealed that in wild-type E9.5 embryos, the nuclear/cytoplasmic intensity ratio is higher in the pDPW compared with the aDPW (Fig. 7c,d), indicative of elevated YAP/TAZ activity in the pDPW. Nuclear YAP/TAZ intensity and the nuclear/cytoplasmic YAP/TAZ intensity ratio are reduced and homogeneous in the DPW at E8 and after OFT transection (Fig. 7d and Supplementary Figs 7 and 8). Moreover, reduced nuclear YAP/TAZ intensity and a reduced homogeneous YAP/TAZ nuclear/cytoplasmic intensity ratio were observed in the DPW of *Nkx2-5*^*−/−*^ embryos (Fig. 7d and Supplementary Fig. 8). Consistent with these results, quantification of cells with cytoplasmic YAP/TAZ confirmed that cytoplasmic YAP/TAZ is significantly more abundant in the aDPW than pDPW at E9.5 (Supplementary Fig. 8). Furthermore, the distribution of cytoplasmic YAP/TAZ is homogeneous at E8, after OFT transection and in *Nkx2.5*^*−/−*^ embryos (Supplementary Figs 7 and 8). The pDPW at E9.5 is thus characterized by elevated proliferation and increased nuclear YAP/TAZ, that coincides spatiotemporally with altered patterns of mechanical stress, including under conditions of experimental and genetic perturbation.

**Figure 8:**
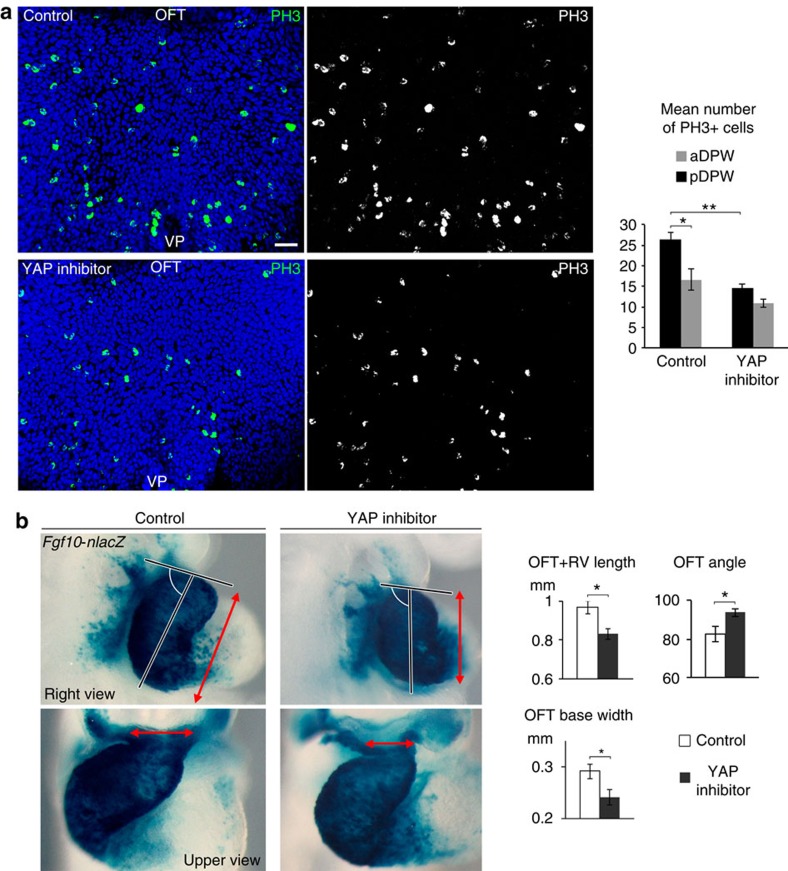
YAP promotes proliferation in the DPW and arterial pole elongation. (**a**) Phospho-Histone H3 staining in the DPW of control embryos and embryos treated with verteporfin, a YAP inhibitor. The plot shows decreased numbers of PH3+ cells in the pDPW after YAP inhibition (Control *n*=5 embryos; verteporfin-treated *n*=8 embryos). (**b**) β-galactosidase expression in the outflow tracts of *Fgf10-nlacZ* transgenic embryos after 24 h of culture. Embryos cultured in the presence of verteporfin form a shorter OFT and right ventricle (OFT+RV length, right view red arrows) and a narrow base of the distal OFT (upper view, red arrows) with a larger angle between the proximal and distal OFT (Control *n*=9 embryos; verteporfin-treated *n*=12 embryos). *P<0.05; **P<0.01 (Unpaired bilateral Mann–Whitney test). Error bars represent s.e.m. Scale bars, 30 μm.

To investigate whether YAP activity functionally mediates proliferation in the DPW, we cultured WT embryos from E8.5 to E9.5 with verteporfin, a YAP inhibitor^36^. Inhibiting YAP significantly reduced proliferation in the DPW epithelium (mainly in the pDPW) as shown by a reduced number of PH3+ cells (Fig. 8a). Furthermore, YAP inhibition impacts on heart morphogenesis and elongation as treated embryos had a shorter and straighter OFT with a narrow distal region (Fig. 8b and Supplementary Fig. 9). The proximal OFT of verteporfin-treated embryos also appeared abnormally dilated (Fig. 8b and Supplementary Fig. 9). Together, these results show that YAP activity positively regulates proliferation in the DPW and is required for normal OFT morphogenesis.

### Oriented cell division and growth in the DPW epithelium

**Figure 9:**
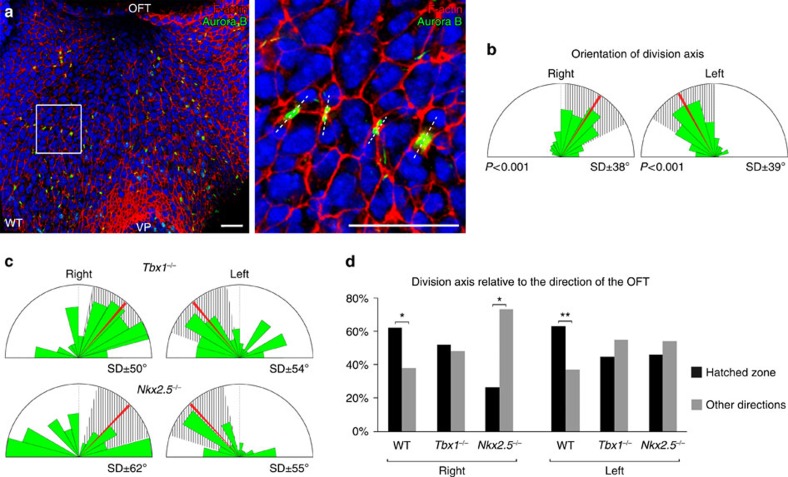
Oriented cell division in the DPW. (**a**) DPW after Aurora B staining showing newly divided cells in the plane of the epithelium. (**b**) Diagram showing daughter cells aligned on an axis directed towards the arterial pole (*n*=7 embryos, right: 172 divisions, left: 215 divisions; *P*-value based on a Rayleigh circular test). (**c**) Daughter cells are more randomly oriented and less directed towards the arterial pole in *Tbx1*^*−/−*^ and *Nkx2-5*^*−/−*^ embryos (*Tbx1*^*−/−*^ *n*=4 embryos, right: 62 divisions, left: 82 divisions; *Nkx2-5*^*−/−*^ *n*=6 embryos, right: 30 divisions, left: 37 divisions). (**d**) Histogram showing that the majority of daughter cells are oriented on an axis directed towards the arterial pole in wild type, but not *Tbx1*^*−/−*^ or *Nkx2-5*^*−/−*^embryos. *P<0.05; **P<0.01 (Chi-squared test). Scale bars, 30 μm.

**Figure 10:**
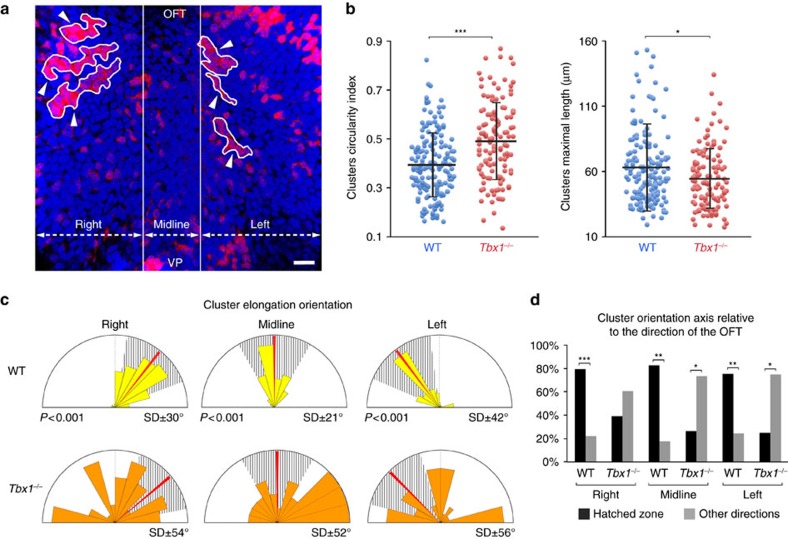
Oriented growth in the DPW. (**a**) Fluorescent labeling of *Mesp1-Cre*;*Rosa-Confetti* cell clusters in the DPW at E9.5, showing isolated RFP-positive cell clusters (white arrowheads) oriented towards the OFT. (**b**) Distribution plots of the circularity index and maximal length of *Mesp1-Cre*;*Rosa-Confetti* cell clusters. In *Tbx1*^*−/−*^ embryos clusters are less elongated and shorter than those in wild-type embryos (*P*-value based on a Rayleigh circular test). (**c**) Diagram showing the axis of elongation of cell clusters in right, midline and left regions relative to the arterial pole (red bar). Cell clusters are more randomly elongated in *Tbx1*^*−/−*^ embryos. (**d**) Histogram showing that cell clusters are elongated on an axis oriented towards the arterial pole in wild type but not *Tbx1*^*−/−*^ embryos. WT *n*=14 embryos, 185 clusters; *Tbx1*^*−/−*^ *n*=9 embryos, 125 clusters. Error bars in **b** represent s.d. *P<0.05; **P<0.01; ***P<0.001 (**b**: Unpaired bilateral *t*-test; **d**: Chi-squared test). Scale bars, 30 μm.

Using Aurora B staining to label nascent daughter cells we investigated whether proliferation in the DPW is oriented in the plane of the epithelium (Fig. 9a). We observed that the distribution of daughter cells within the plane of the epithelium is tightly oriented on an axis directed towards the arterial pole, coincident with that of cell elongation (Fig. 9a,b,d) and consistent with studies showing that mechanical stretch can orient epithelial cell division^23^,^24^,^37^,^38^. The orientation of cell division in the DPW is significantly more random in *Tbx1*^*−/−*^ and *Nkx2.5*^*−/−*^ embryos (Fig. 9c,d), consistent with altered patterns of mechanical stress in these mutants. Oriented cell division can in turn orient the growth of an epithelium^23^,^24^. We generated labelled clusters of cells in the DPW using *Mesp1-Cre* and a *Rosa-Confetti* conditional reporter (Fig. 10a, Supplementary Fig. 10). *Mesp1* is expressed in a short time-window early in development^39^,^40^ and *Mesp1-Cre* labelling results in clusters of cells in the DPW. In contrast, more fragmented labeling was observed when *Confetti* clones were induced by *Mef2cAHF-Cre* which is continuously expressed in the DPW^41^ (Supplementary Fig. 10). Morphometric analysis revealed that isolated cell clusters in the DPW of *Mesp1-Cre*/*Rosa-Confetti* embryos at E9.5 are anisotropic and oriented on an axis directed towards the arterial pole of the heart (Fig. 10b–d, Supplementary Fig. 10). In contrast, clusters analysed at E8 were rounder and those that were elongated were oriented on the embryonic left-right axis, coherent with addition of cells to the early heart tube across the dorsal mesocardium (Supplementary Fig. 10). Analysis of *Tbx1*^*−/−*^ embryos at E9.5 revealed that *Mesp1-Cre*/*Rosa-Confetti* labelled clusters are more compact, less elongated and more randomly oriented compared with those in wild-type embryos, consistent with altered patterns of cell deformation, proliferation and division axis (Fig. 10b–d, Supplementary Fig. 10). These results suggest that failure to orient cell division underlies epithelial disorganization in the *Tbx1*^*−/−*^ DPW and that oriented epithelial growth contributes to SHF deployment.

## Discussion

The epithelial properties of SHF cells are emerging as a regulatory step in heart tube elongation. In this study we present evidence that SHF cells in the DPW are subject to mechanical forces resulting in oriented epithelial tension. First, cells in the pDPW are elongated in the direction of the arterial pole of the heart. Second, analysis of wound and surrounding cell shape changes after epithelial wounding indicates that cells in the pDPW are subject to anisotropic mechanical forces. Cells in the pDPW appear to be subject to higher levels of mechanical stress. Third, polarized junctional tension in pDPW cells, revealed by active actomyosin complex formation, is aligned with the direction of cell elongation. Further cell elongation on ROCK inhibitor treatment suggests that polarized actomyosin distribution is a response to tissue stretching by extrinsic forces. Similar patterns of orientated cell elongation and polarized junctional tension have been documented in *Drosophila* in response to hinge contraction and anisotropic epithelial forces in the pupal wing and during growth of the wing disc^21^,^23^,^24^,^42^. While our results are consistent with elevated epithelial tension in the pDPW, we are currently unable to directly measure tension in the DPW due to technical limitations in analysing the physical properties of splanchnic mesoderm in living embryos.

The onset of cell elongation, oriented mechanical force and polarized distribution of actomyosin in the pDPW coincides temporally with the deployment of epithelial progenitor cells to the poles of the heart tube. Furthermore, cell elongation and polarized actomyosin accumulation require contiguity with an intact heart tube and are perturbed in *Tbx1* and *Nkx2-5* mutants in which SHF deployment is defective. These results implicate progenitor cell addition to the poles of the heart tube as a source of mechanical stress in the DPW. Additional sources cannot be discounted and potentially include cardiac contraction as well as tensile forces within underlying pharyngeal endoderm, implicated in earlier stages of heart tube formation^43^. Myocardial tissue deformations have also been recently reported during early heart tube assembly^44^. Compressive forces during growth of the venous pole may also contribute to cell deformation in the DPW. Oriented cell elongation and polarized actomyosin are predominantly observed in the pDPW, suggesting greater sensitivity to cell deformation. Indeed, increased basal lamina formation and epithelial cohesion due to the presence of E-cadherin and absence of Wnt5a expression has been reported in the aDPW as SHF cells approach the arterial pole of the heart^12^,^20^.

Mechanical signals and tissue-level forces can generate form by coordinating cell movements, regulating proliferation and orienting cell division^2^. Our results suggest that mechanical forces and epithelial stress in the DPW impact on the rate and orientation of cell division and consequently on progenitor cell deployment. Epithelial tension in the pDPW may thus be a component of a biomechanical feedback process driving terminal extension of the heart tube. Nuclear YAP/TAZ couples epithelial cell deformation and mechanical cues with the regulation of proliferation and differentiation^31^ and accumulates in the pDPW. Blocking YAP activity in embryo culture results in impaired proliferation in the DPW and defective arterial pole development. YAP is required in the cardiac crescent for embryonic cardiomyocyte proliferation, however the specific requirements of YAP and TAZ in the SHF remain to be investigated by conditional mutagenesis^45^. Newly divided daughter cells in the DPW are oriented along the axis of cell elongation, consistent with cell division patterns in stretched epithelial monolayers^46^. Furthermore, our analysis of labelled cell clusters reveals oriented patterns of growth in the DPW directed towards the arterial pole of the heart. Similarly, global tension patterns orient cell stretching patterns and cell division to determine tissue shape in the *Drosophila* wing imaginal disc^2^,^24^. Our results implicate tissue-level forces in SHF development, providing an additional example of how mechanical forces guide cardiogenesis^43^,^44^,^47^,^48^. Dissecting the mechanisms by which epithelial tension impacts on heart tube elongation, including the influence of embryonic laterality, will provide insights into how mechanical feedback regulates vertebrate organogenesis as well as into the origin of congenital heart defects.

## Methods

### Experimental animals

The following mouse lines were used: *Tbx1*^*+/−*^(ref. 49), *Nkx2.5*^*+/−*^ (ref. 28), *Mesp1-Cre*^39^, *Mef2c-AHF-Cre*^41^*, Rosa-Confetti*^50^, *Mlc1v-nlacZ-24* (*Fgf10-nlacZ* enhancer trap line)^51^ and CD1 mice. *Mesp1-Cre* and *Mef2c-AHF-Cre* mice were crossed to *Rosa-Confetti* mice to generate labelled cell clusters in the DPW. Mice were maintained on a mixed C57Bl/6 and CD1 background. Animal experiments were carried out in agreement with national and European law and approved by the Ethics Committee for Animal Experimentation of Marseille and the French Ministry for National Education, Higher Education and Research.

### Scanning electron microscopy

E9.5 embryos were dissected and hearts were removed. Embryos were fixed in 2.5% glutaraldehyde in PBS for 3 h at room temperature. Embryos were post-fixed in 1% OsO4 in PBS for 2 h at 4 °C and progressively dehydrated in ethanol. Embryos were then dessiccated in ethanol/hexamethyldisilizane (sequential 2/1, 1/1, 1/2 ratios) followed by pure hexamethyldisilizane. Embryos were gold metallized and visualized with a Leica S440.

### Embryo dissection and immunostaining

Embryos were fixed for 1 h in 4% paraformaldehyde (PFA) for routine immunostaining, 8% PFA for phosphorylated myosin staining and overnight in 2% PFA for visualization of Rosa-Confetti clones. After fixation, the heart was removed to expose the dorsal pericardial wall and embryos processed for whole mount staining. For immunostaining, embryos were blocked overnight in blocking buffer (PBS/0,1% Triton/3% BSA), incubated overnight at 4 °C with primary antibodies, washed in blocking buffer and incubated overnight at 4 °C with species-specific secondary antibodies. Finally, embryos were stained for F-actin with Phalloidin-TRITC and/or counterstained with Hoechst. For analysis of *Rosa-Confetti* embryos Phalloidin-Atto 647 or Hoechst counterstain was used. After staining, the pharyngeal region was microdissected and the dorsal pericardial wall imaged ventrally using a confocal LSM 780 microscope. Confocal images were acquired as Z-stacks.

The following antibodies were used: rabbit anti-ppMLC (1/200, Cell Signaling Thr18/Ser19 3674), mouse anti-pMLC (1/200, Cell Signaling Ser19 3675), rabbit anti-Phospho Histone H3 (1/400, Upstate Cell Signaling 06-570), mouse anti-YAP (1/100, Santa Cruz sc-101199), mouse anti-Aurora B (1/200, BD Transduction Laboratories 611082), goat anti-Nkx2.5 (1/100, Santa Cruz sc-8697). Fluorescent secondary antibodies Alexa 488, 568 and Cy5 from Jackson and Invitrogen were used at 1/500. Two fluorescent-conjugated forms of Phalloidin were used to label F-actin: Phalloidin-TRITC (1/50, Sigma P1951), Phalloidin-Atto 647 (1/200, Sigma 65906).

### Segmentation analysis and polarity and shape quantification

The apical surface of the DPW epithelium was imaged in a ventral view. The apical cortical F-actin belt was used as an apical membrane marker for segmentation. Segmentation of the epithelium was performed using Tissue Analyser software developed by Benoît Aigouy^21^. The software is an ImageJ plugin using a watershed algorithm to segment the cell cortex. Briefly, base segmentation was automatically performed by the Tissue Analyser software and verified and corrected manually. Apical surface area heatmap, cell elongation axis representation and nematic bars representing protein polarity and cell elongation axis were generated using the same software. Segmentation data were imported into ImageJ to calculate apical surface area, length-to-width ratio and angle of orientation relative to the embryonic anterior posterior axis. Segmentation was either performed on the entire DPW or on cells in randomly positioned quadrants in the anterior, posterior, left or right regions of the DPW. Rose circular diagrams were generated using MatLab with a bin of 15° to represent the cell elongation axis. Mean direction towards the OFT were imposed on the rose plots and the percentage of cells elongated in a +/−30° radius around OFT direction was calculated to investigate a possible significant focus of elongation axis. Segmentation was performed on three or more embryos of each of the different developmental stages, genotypes or manipulations, as follows: Apical surface area and length-to-width ratio: wild-type E9.5 *n*=3 embryos, pDPW: 1,522 cells, aDPW: 1,688 cells; E8 pDPW *n*=5 embryos, 468 cells; OFT cut *n*=8 embryos, 637 cells; *Tbx1*^*−/−*^ *n*=3 embryos, pDPW: 657 cells, aDPW: 455 cells; *Nkx2-5*^*−/−*^ pDPW *n*=6 embryos, 356 cells; Y27632 treatment *n*=7 embryos, right: 265 cells. Angle of cell elongation: wild-type E9.5 *n*=3 embryos, right: 1,462 cells, left 1,671 cells; E8 pDPW *n*=5 embryos, 468 cells; OFT cut *n*=8 embryos, 637 cells; *Tbx1*^*−/−*^ *n*=3 embryos, right: 388 cells, left 516 cells; *Nkx2-5*^*−/−*^ pDPW *n*=6 embryos, right: 201 cells, left: 155 cells.

### Mechanical stress assessment with a wound assay

Circular wounds of 15–30 μm diameter were made on the right side of the pDPW and aDPW epithelium of living embryos using drawn glass pipettes and the embryos were fixed after 2–5 min (see embryo culture section). After Phalloidin staining and confocal imaging, the perimeter of the wounds were drawn manually in ImageJ and length to width ratio and mean elongation axis of the wounds were quantified. On high magnification, the apical membranes of cells surrounding wounds generated in the right side of the pDPW and aDPW of living embryos (see embryo culture section) were manually drawn in ImageJ and length to width ratio and elongation axis were quantified. To assess cell shape changes subsequent to wounding, cell morphology around the wound was quantified and cells separated in four populations. Cells directly adjacent to the wound, including cells lateral to the wound on the left and right sides (indicated with red asterisks in Fig. 2), and cells present above and below the wound (green asterisks in Fig. 2); and cells away from the wound, lateral and above/below but 3 to 4 cell rows away from the wound (yellow and light blue asterisks, respectively). Cell Length to Width ratios were quantified for each population and compared with control cells measured on the left side of the DPW of each manipulated embryo. Cell elongation axis angles were measured and Rose circular diagrams were generated using MatLab with a bin of 15°. The percentage of cells oriented in an axis +/−30° around the direction of the OFT was calculated and compared with the cell elongation axis in contralateral control cells from the respective regions. Cells around wounds were also segmented using Tissue Analyser software and isolated and colour coded to better appreciate cell morphology. Cell elongation nematic bars were created using Tissue Analyser software to give both the axis of elongation (bar angle) and amplitude of elongation (bar length). Bean plot representations of length to width ratio distribution were generated using R software. Wound length to width ratio was measured for the following numbers of embryos: WT E9.5 pDPW: *n*=22; WT E9.5 aDPW: *n*=11; WT E8 pDPW: *n*=6; Nkx2-5 E9.5 pDPW: *n*=5. Cell shape changes around the wound were analysed for the following numbers of embryos: WT E9.5 pDPW: *n*=11 embryos, 65 red cells, 57 yellow cells, 61 green cells, 59 light blue cells and 69 control cells; WT E9.5 aDPW: *n*=4 embryos, 18 red cells, 32 yellow cells, 21 green cells, 20 light blue cells and 29 control cells. WT E8 pDPW; *n*=5 embryos, 22 red cells, 22 green cells and 33 control cells. *Nkx2-5*^*−/−*^ E9.5 pDPW; *n*=5 embryos, 25 red cells, 28 green cells and 43 control cells.

### Proliferation and orientation of cell division measurements

Proliferation heat maps were generated by counting Phospho-HistoneH3-positive cells in the DPW epithelium that were mapped onto a schematic DPW comprised of an 8 by 8 grid of small sub-regions. Data from multiple embryos were merged in one schematic DPW and a colour code imposed to illustrate the % of PH3+ positive cells present in each region.

The orientation of cell division was analysed using ImageJ software following Aurora B staining to detect newly divided daughter cells. The angle of the axis between the two nuclei of newly divided daughter cells passing through the Aurora B positive junction was measured relative to the embryonic anterior posterior axis. Rose circular diagrams were generated using MatLab with a bin of 15°.

The following numbers of embryos were analysed. Proliferation: wild-type E9.5 *n*=9 embryos; E8 *n*=3 embryos; *Tbx1*^*−/−*^ *n*=4 embryos. Division axis: wild-type E9.5 *n*=7 embryos, right: 172 divisions, left: 215 divisions; *Tbx1*^*−/−*^ *n*=4 embryos, right: 62 divisions, left: 82 divisions; *Nkx2-5*^*−/−*^ *n*=6 embryos, right: 30 divisions, left: 37 divisions.

### YAP activity measurement and inhibition

For YAP/TAZ intensity measurement, 3 to 5 μm stack projections were generated for small anterior and posterior regions of the DPW. Nuclei were then segmented and nuclear and non-nuclear mean YAP/TAZ intensity per area were quantified to calculate the nuclear/non-nuclear ratio. Cytoplasmic YAP/TAZ heat maps were generated by counting and localizing cells with cytoplasmic YAP/TAZ in the DPW epithelium and processed as for proliferation heat maps. Wild-type E9.5 *n*=6 embryos; OFT cut E9.5 *n*=8 embryos; *Nkx2-5*^*−/−*^ E9.5 *n*=7 embryos; wild-type E8 *n*=5 embryos.

For YAP inhibition, E8.5 embryos were cultured with verteporfin (see embryo culture section). PH3 staining was carried out on control and treated embryos and PH3+ cells counted throughout the DPW to evaluate posterior/anterior differences (Control *n*=5 embryos; verteporfin-treated *n*=8 embryos). Wild-type and *Fgf10-nlacZ* transgenic mice were treated to evaluate the impact of YAP inhibition on arterial pole morphogenesis. OFT and right ventricular length and the angle between the distal and proximal regions of the OFT were measured on right lateral views. Distal OFT base width was measured on ventral/superior views of dissected embryos (Control *n*=9 embryos; verteporfin-treated *n*=12 embryos).

### *Rosa-Confetti* clone analysis

After image acquisition, labelled cell clusters in *Rosa-Confetti* embryos were isolated and manually drawn. ImageJ was used to calculate cluster circularity index, maximal length and mean orientation angle. Rose circular diagrams were generated using MatLab with a bin of 15°.

Clusters circularity index and maximal length: wild-type *n*=14 embryos, 185 clusters; *Tbx1*^*−/−*^ *n*=9 embryos, 125 clusters. Clusters orientation angles: wild type right: 73 clusters, middle: 40, left: 72; *Tbx1*^*−/−*^ right: 51 clusters, middle: 34, left: 40.

### Embryo culture

E8.5 embryos were dissected in CO_2_ independent medium (Invitrogen, 18045-054), 0.04% BSA (Albumin, from bovine serum, SIGMA A88056), 1% penicillin/streptomycin (Invitrogen, 15140-122) without damaging the yolk sac. Embryos were cultured in rolling-bottles for 24 h in 75% rat serum (Harlan SR-0100 or IGBMC Strasbourg), 25% DMEM (Invitrogen) and 1% penicillin/streptomycin. For the first 12 h of culture embryos were exposed to a mixture of 5% CO_2_, 5% O_2_, 90% N_2_ and subsequently switched to 5% CO_2_, 20% O_2_, 75% N_2_. For Myosin2 inhibition, E9.5 embryos were cultured for 40 min with the ROCK inhibitor Y-27632 at 30μM (Enzo life sciences, ALX-270-333).

For the OFT transection experiment, E9.5 embryos were cultivated for 3 h. Embryos were removed from the yolk sac, which was left attached to the embryo. A small opening was generated on the right side of the pericardium and the OFT was sectioned. In control embryos a similar opening was generated in the pericardium without sectioning the OFT.

For the DPW wounding experiment, E9.5 embryos were removed from the yolk sac and a circular wound generated on the right side of the DPW in the posterior or anterior region using a fine drawn glass pipette. Embryos were maintained at 37 °C for 2–5 min before fixation.

For YAP inhibition, E8.5 embryos were cultured for 24 h with verteporfin (Sigma, SML0534) at 20 μmol l^−1^ and control embryos cultured in parallel with dimethylsulphoxide.

### Statistics

Quantification was performed on cells from a minimum of three embryos for each result. Embryos in which immunostaining failed or which were damaged were excluded from the samples. Appropriate statistical tests were used for each sample. No randomization or blinding was used. Statistical analysis of data concerning apical surface area, length-to-width ratio, intensity quantification, proliferation, circularity index and maximal length of clusters were performed using an Unpaired bilateral Mann–Whitney test for small samples (*n*<100) or a *t*-test for larger samples (*n*>100). Statistics on percentages were done using a Chi-squared test. Statistics on angular distribution were performed using a Rayleigh circular test.

### Data availability

The authors declare that all data supporting the findings of this study are available within the article and its Supplementary Information files or from the corresponding author upon reasonable request.

## Additional information

**How to cite this article:** Francou, A. *et al*. Epithelial tension in the second heart field promotes mouse heart tube elongation. *Nat. Commun.* **8,** 14770 doi: 10.1038/ncomms14770 (2017).

**Publisher's note**: Springer Nature remains neutral with regard to jurisdictional claims in published maps and institutional affiliations.

## Supplementary Material

Supplementary Information Supplementary Figures.
