# Supplementary material for: Epithelial tension in the second heart field promotes mouse heart tube elongation
Source: Nat Commun. 2017 Mar 30;8:14770. doi: 10.1038/ncomms14770 (PMC5379109; doi:10.1038/ncomms14770)
Supplement: Supplementary Information — Supplementary Figures. [file ncomms14770-s1.pdf]

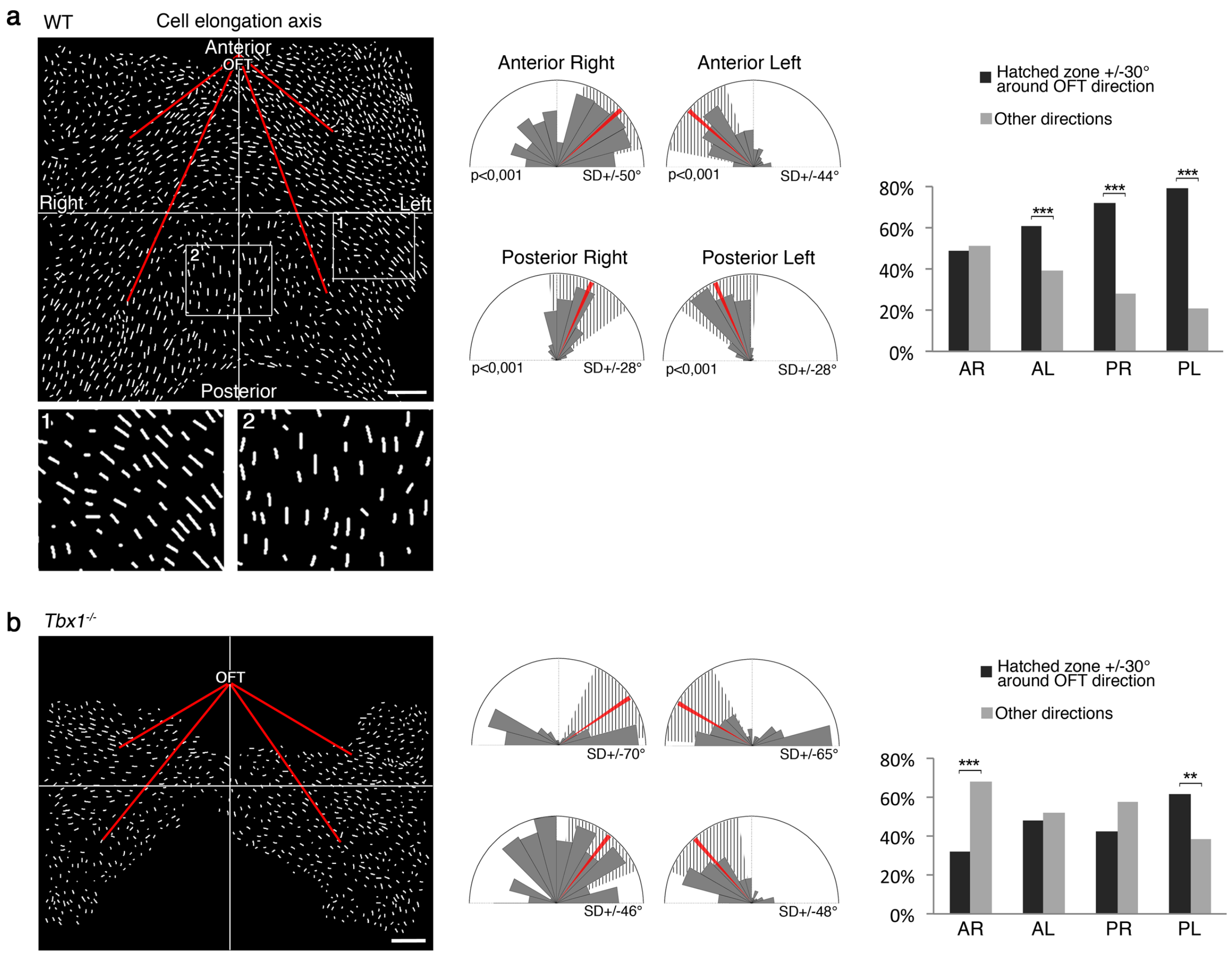

Supplementary Figure 1. Determination of the cell elongation axis in the DPW. (**a**) Representation of the elongation axis of each cell in the epithelium segmented in Figure 1 shows that cells tend to be elongated on an axis directed towards the OFT in lateral (1) and midline (2) regions (high magnification box). Here four regions are defined in the epithelium. Rose circular diagrams and histograms showing that the cell elongation angle in wildtype embryos is convergent in each region and that the majority of cells are elongated on an axis directed towards the OFT (red lines represent the mean OFT direction per region) (P value based on a Rayleigh circular test). (**b**) In *Tbx1*$^{-/-}$ embryos the angle of cell elongation is not oriented across the DPW and only a small proportion of cells are elongated on an axis directed towards the OFT, in particular in the aDPW. **, P<0.01; ***, P<0.001 (Chi squared test). Scale bars: 30μm.

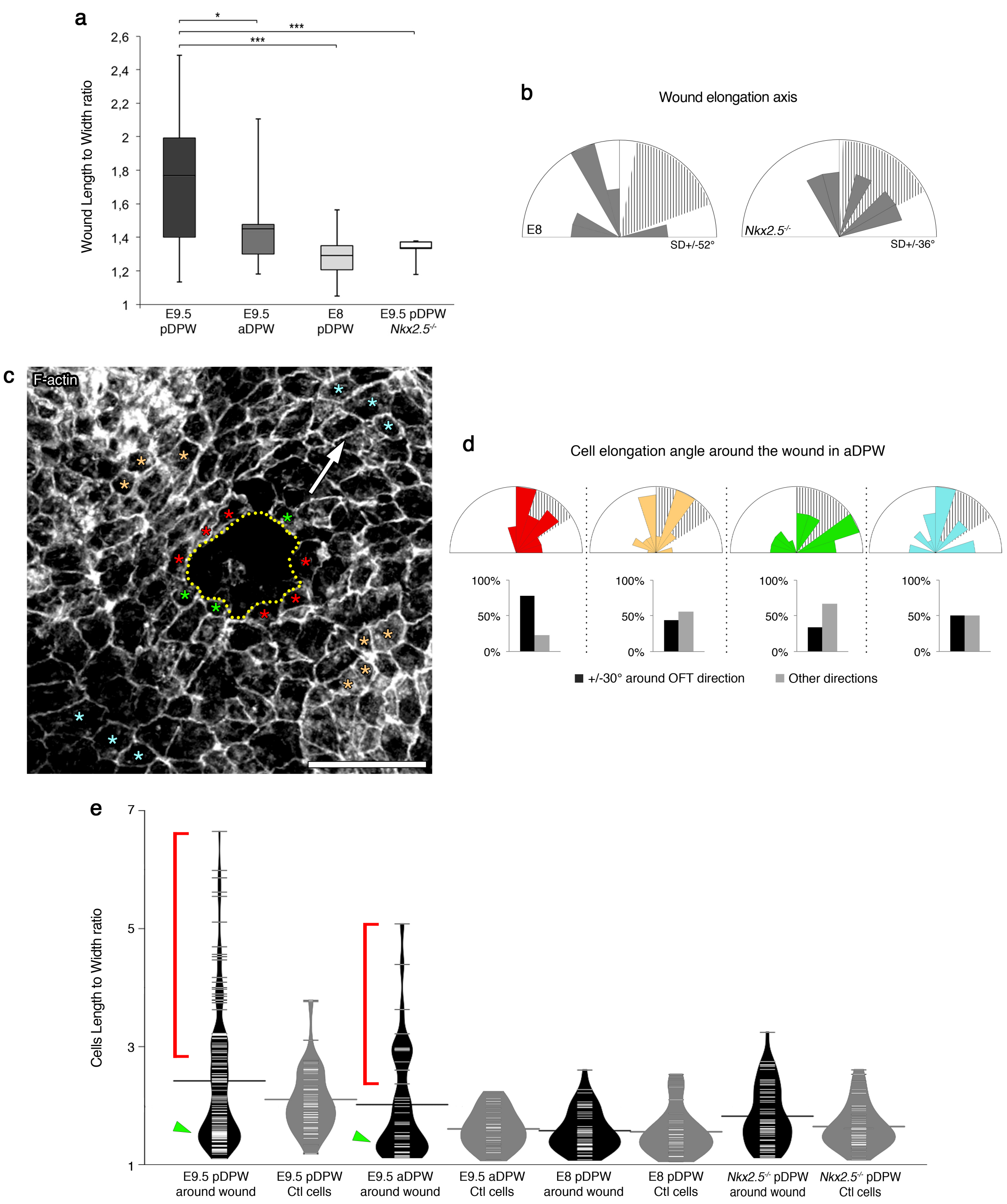

Supplementary Figure 2. Wound assay in the DPW of living embryos. (**a**) Analysis of embryos fixed 2-5 minutes after wounding showing that wounds are elongated in the pDPW of E9.5 embryos compared to the aDPW and the pDPW of E8 and *Nkx2-5*$^{-/-}$ embryos (determined by the length to width ratio) (E9.5 pDPW: n = 22 embryos; E9.5 aDPW: n = 10 embryos; E8 pDPW: n = 6 embryos; *Nkx2-5*$^{-/-}$ E9.5 pDPW: n = 5 embryos). (**b**) Wounds are not significantly elongated on an axis directed towards the OFT in the E8 pDPW or pDPW of *Nkx2-5*$^{-/-}$ embryos. (**c**) Example of a wound in the aDPW of an E9.5 embryo and cell

elongation axis quantification around the wound region in the aDPW (**d**). (**e**) Bean plot showing the L/W distribution of cells around wounds (black) and control cells (grey). In E9.5 pDPW and aDPW control cells, the L/W ratio is distributed compactly around the mean, whereas around wounds the distribution is spread and we can distinguish two populations with low (green arrowheads) and high (red bracket) L/W ratios corresponding to cells anterior/posterior and lateral to the wound respectively. In the pDPW of WT E8 and *Nkx2-5*$^{-/-}$, L/W ratio distributions around wounds tend to be compact around the mean as in control cells (small white and black horizontal lines represent measurements of single cells, long grey horizontal lines represent the mean of each population). In the box plot, the center lines show the median, box limits show the first and third quartiles, and whiskers show maximum and minimum values. *, P<0.05; **, P<0.01; ***, P<0.001 (Unpaired bilateral Mann-Whitney test). Scale bars: 30μm.

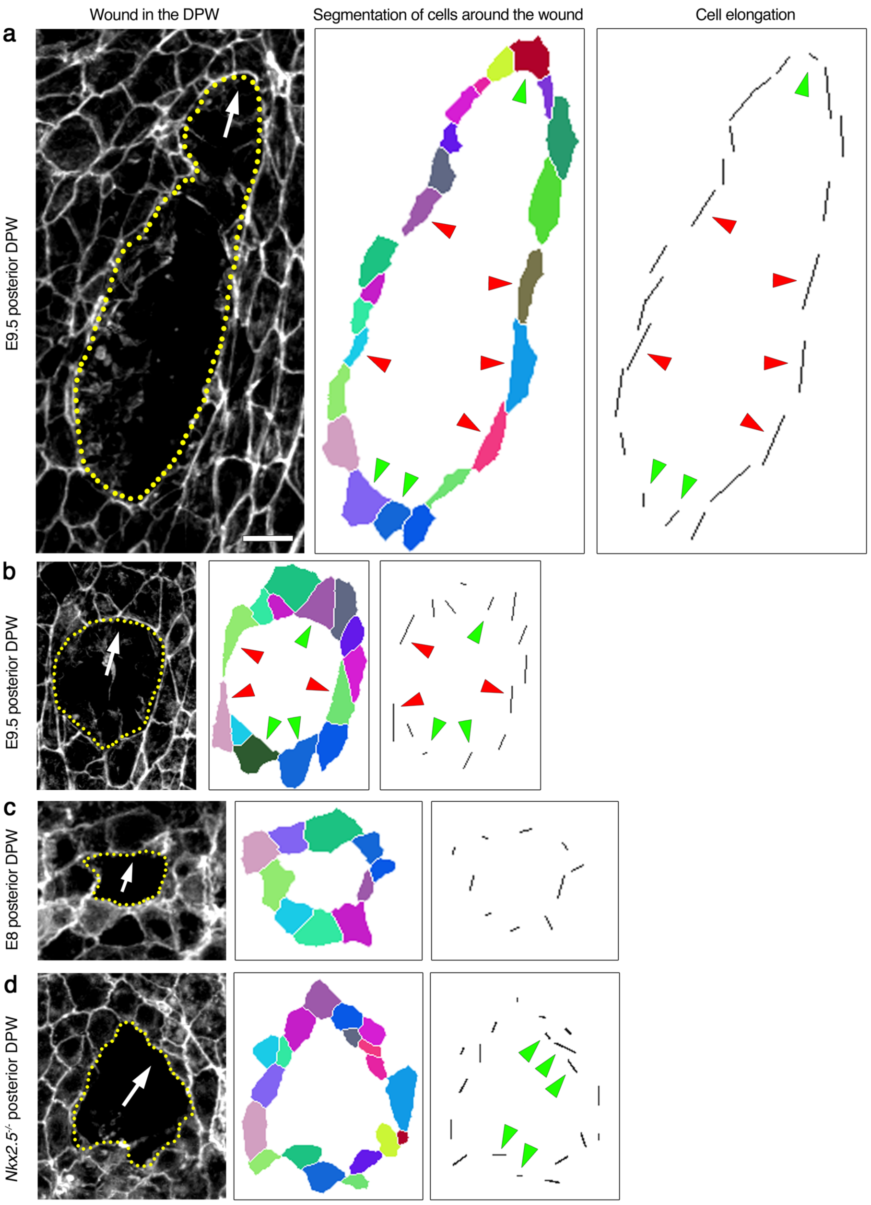

Supplementary Figure 3. Cell morphology around wounds in the DPW. (**a, b**) Wounds in the posterior DPW of E9.5 WT embryos. White arrows show the direction of the arterial pole. Cells around the wound are segmented and coloured to visualise cell shape. Cell elongation is shown by nematic bars indicating the axis of elongation and the amplitude (bar length). Cells lateral to the wounds are highly elongated and oriented on an axis towards the arterial pole (red arrowheads), while cells anterior and posterior to the wound are less elongated. (**c**) In the pDPW of E8 WT embryos, cells around the wound are not elongated or orientated. (**d**) In the pDPW of *Nkx2-5*$^{-/-}$ embryos, cells around the wound are mildly elongated and tend to elongate around the wound, parallel to the wound border. Scale bars: 10μm.

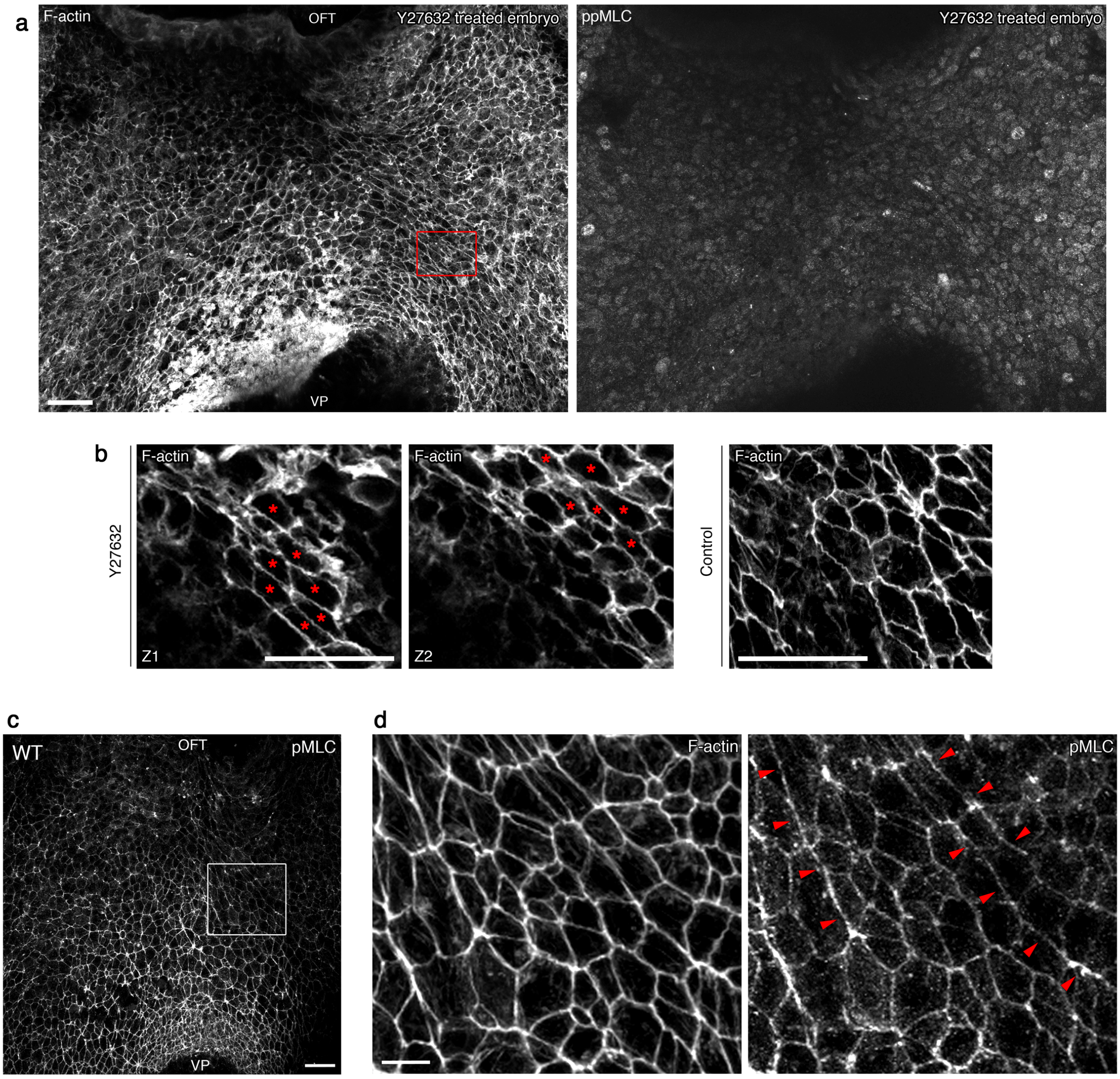

Supplementary Figure 4. ROCK inhibition and monophosphorylated myosin (pMLC2) in the pDPW. (**a**) Whole embryos were treated with the ROCK inhibitor Y27632 to suppress Myosin activity (ppMLC2) in the DPW. (**b**) After Y27632 treatment cell elongation increases (red asterisks, high magnification from a) although global changes in the DPW are not observed over the time of culture. Quantification revealed a significant increase in cell elongation in treated compared to control embryos (see Fig. 2f). (**c**) Monophosphorylated myosin light chain accumulates in the pDPW. (**d**) pMLC2 appears less polarised than ppMLC2 however cables spanning several elongated cells can be observed (red arrowheads). Scale bars: a,c: 30μm; b,d: 10μm.

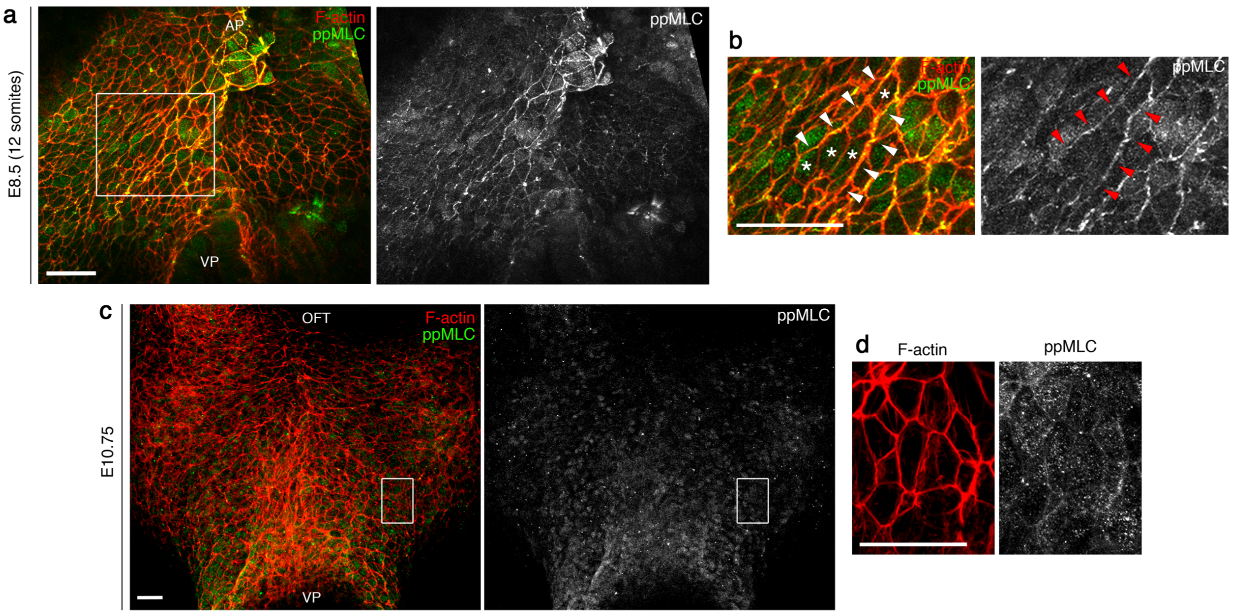

Supplementary Figure 5. Temporal dynamics of actomyosin distribution during heart tube elongation. (**a**) Ventral view of an E8.5 embryo (12 somites), after the dorsal mesocardium has started to break down, showing ppMLC2 labelling. (**b**) High magnification of the boxed area in a, showing cells stretched toward the arterial pole (white asterisks) and formation of myosin cables (arrowheads). (**c**) Myosin staining at E10.75 when heart tube elongation is complete, showing extremely low levels of ppMLC2 accumulation in the epithelium. (**d**) High magnification of the boxed region in c. AP: arterial pole, OFT: outflow tract, VP: venous pole. Scale bars: 30μm.

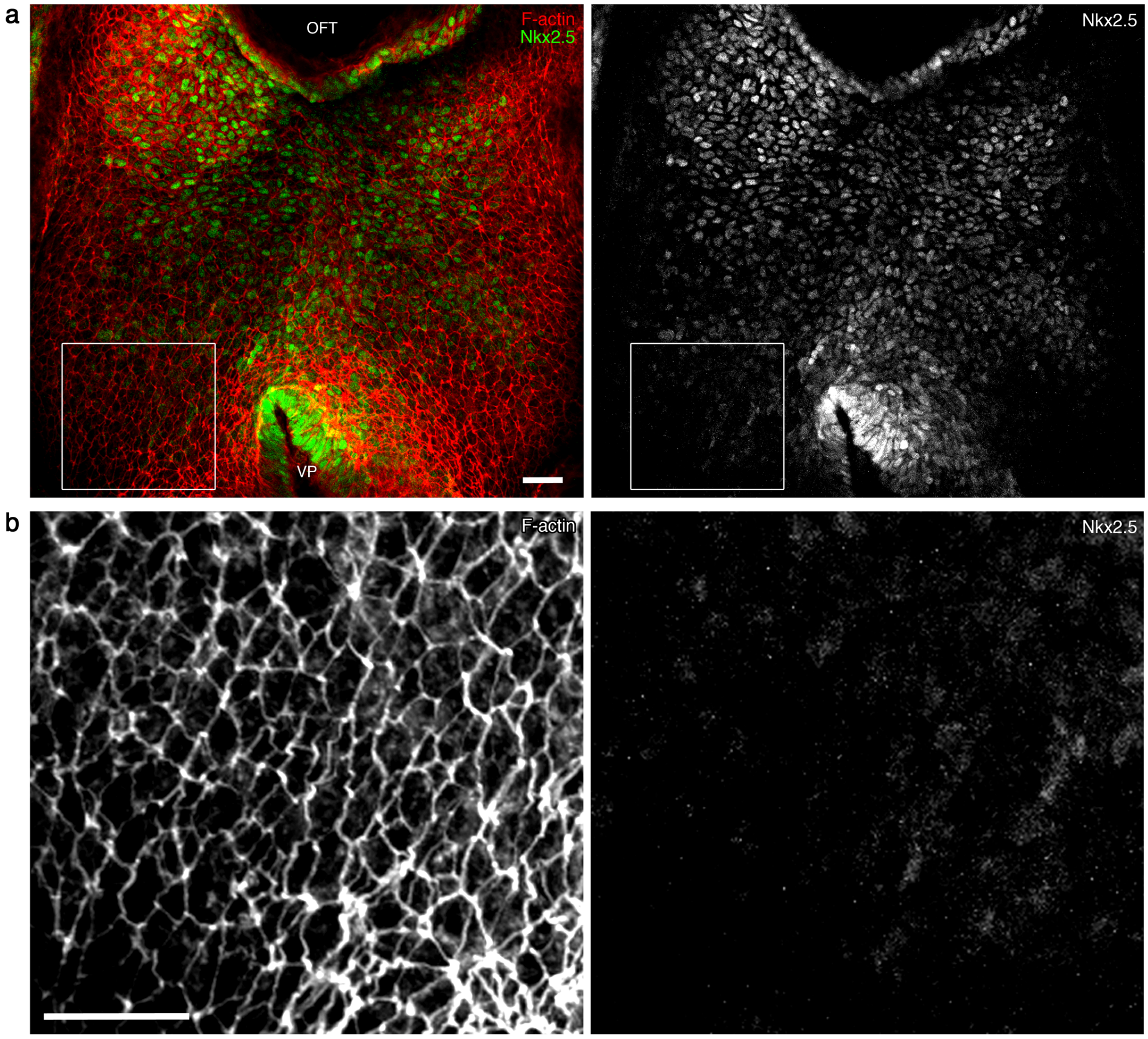

Supplementary Figure 6. Nkx2-5 expression in the DPW epithelium. (**a**) Ventral view of the DPW after immunofluorescence showing Nkx2-5 expression (green) preferentially in the anterior and central region of the epithelium. (**b**) High magnification (boxed in a) of a region of the pDPW where Nkx2-5 is not expressed but where cell elongation and epithelial tension are observed. AP: arterial pole, OFT: outflow tract, VP: venous pole. Scale bars: 30μm.

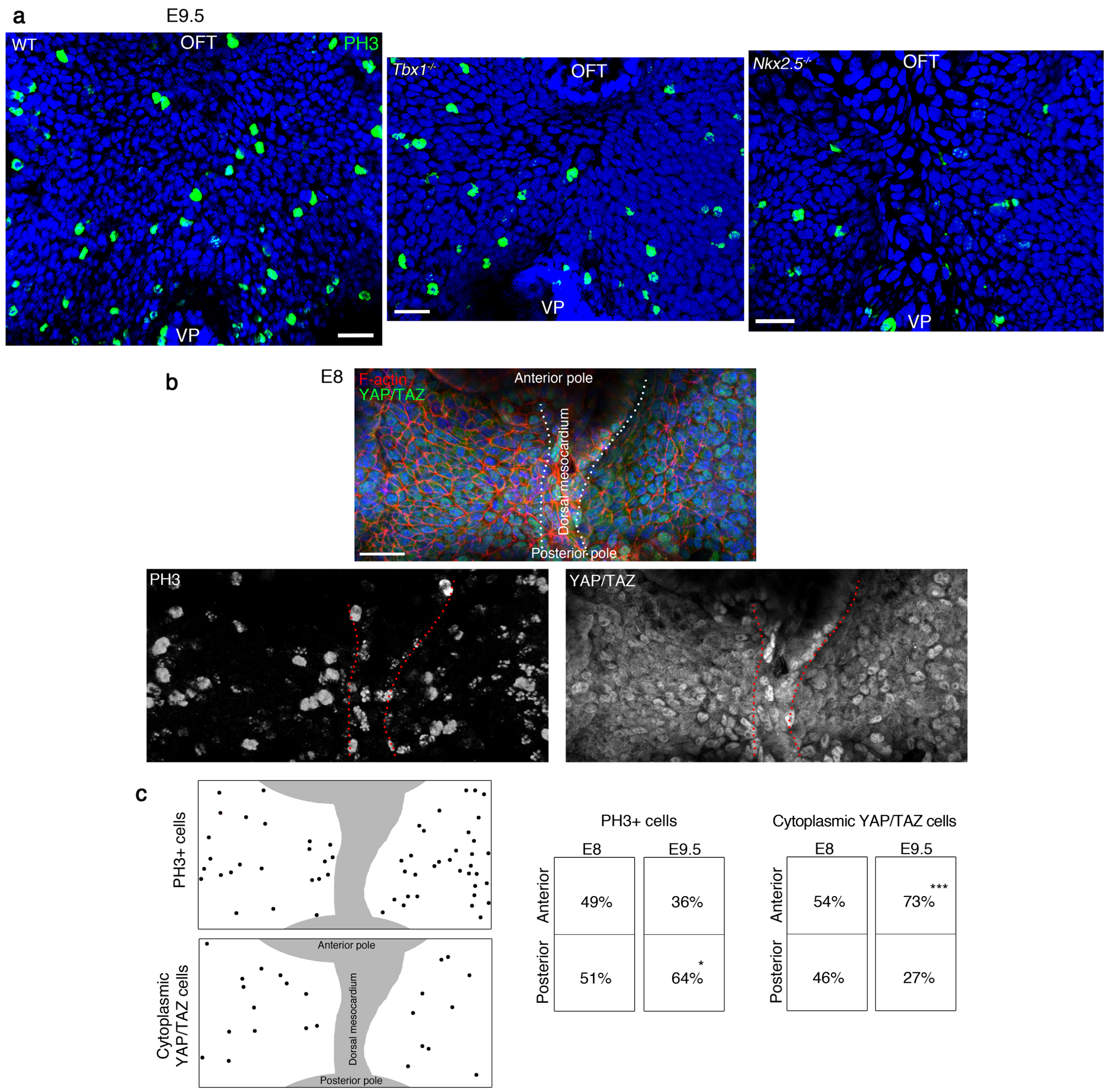

Supplementary Figure 7. PH3+ cells in *Tbx1*$^{-/-}$ and *Nkx2-5*$^{-/-}$ embryos and proliferation and YAP/TAZ distribution at E8. (**a**) **Phospho Histone H3** (PH3) staining showing a reduced number of positive cells in *Tbx1*$^{-/-}$ and *Nkx2-5*$^{-/-}$ embryos compared to wildtype embryos. (**b**) PH3 and YAP/TAZ staining in the DPW epithelium of an E8 embryo. (**c**) The distribution of PH3+ cells and cytoplasmic YAP/TAZ cells at E8 showing an equivalent distribution in anterior and posterior regions (PH3, E8, n=3; E9.5: n=9; YAP/TAZ, E8: n=3, E9.5: n=6; PH3, E8, n=3; E9.5: n=9). *, P<0.05; ***, P<0.01 (Chi squared test). Scale bars: 30μm.

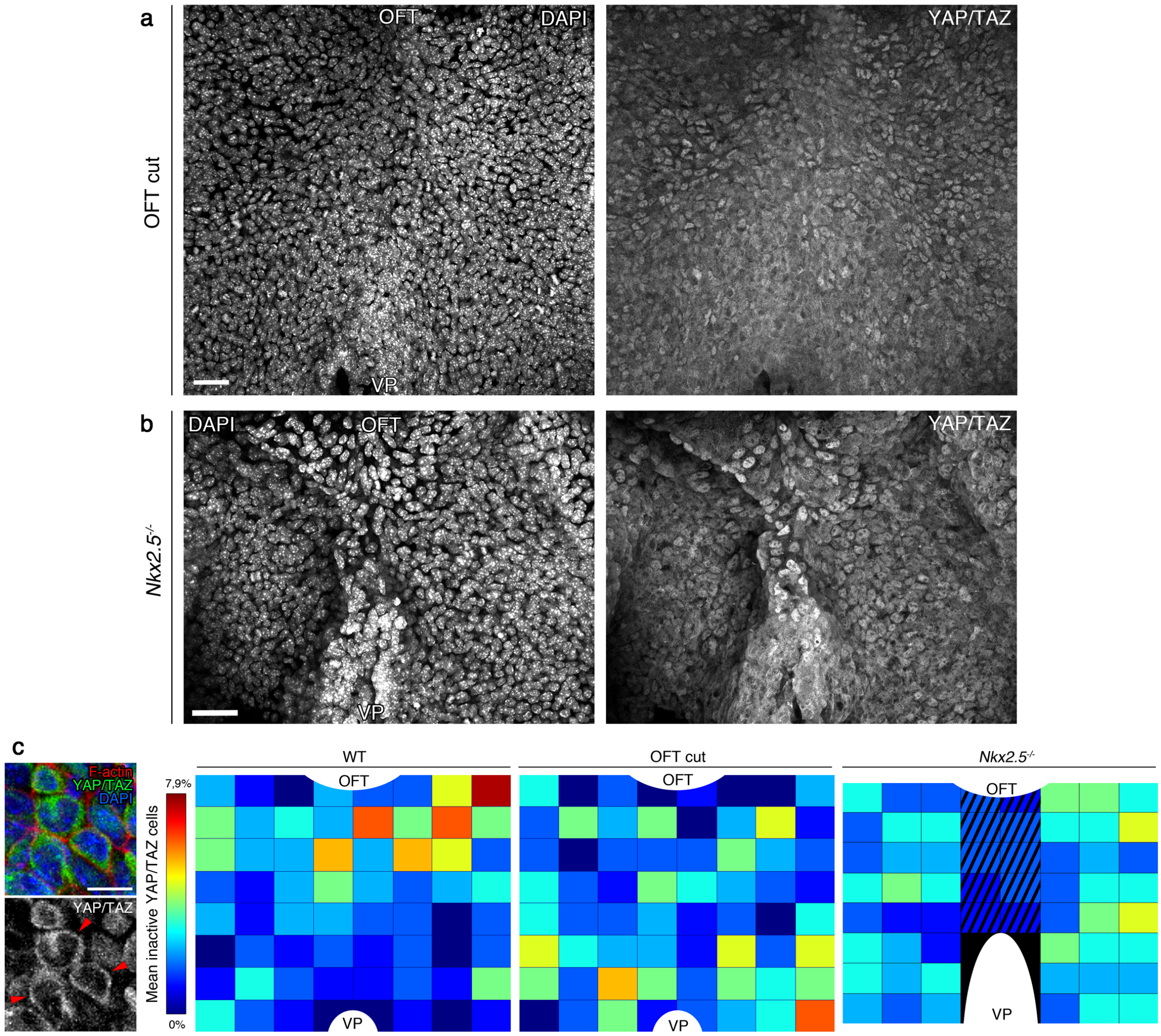

Supplementary Figure 8. YAP/TAZ distribution after OFT transection and in *Nkx2-5*$^{-/-}$ embryos. YAP/TAZ staining in an embryo after heart transection and embryo culture (**a**) and in an *Nkx2-5*$^{-/-}$ embryo (**b**) showing reduced nuclear staining compared to wildtype (Fig. 7b). **(c)** High magnification view showing cytoplasmic YAP/TAZ labelling. Heat maps showing a higher incidence of cytoplasmic YAP/TAZ labelling in the aDPW of wildtype embryos but not after OFT transection or in *Nkx2-5*$^{-/-}$ embryos. Scale bars: a,b: 30μm; c: 10μm.

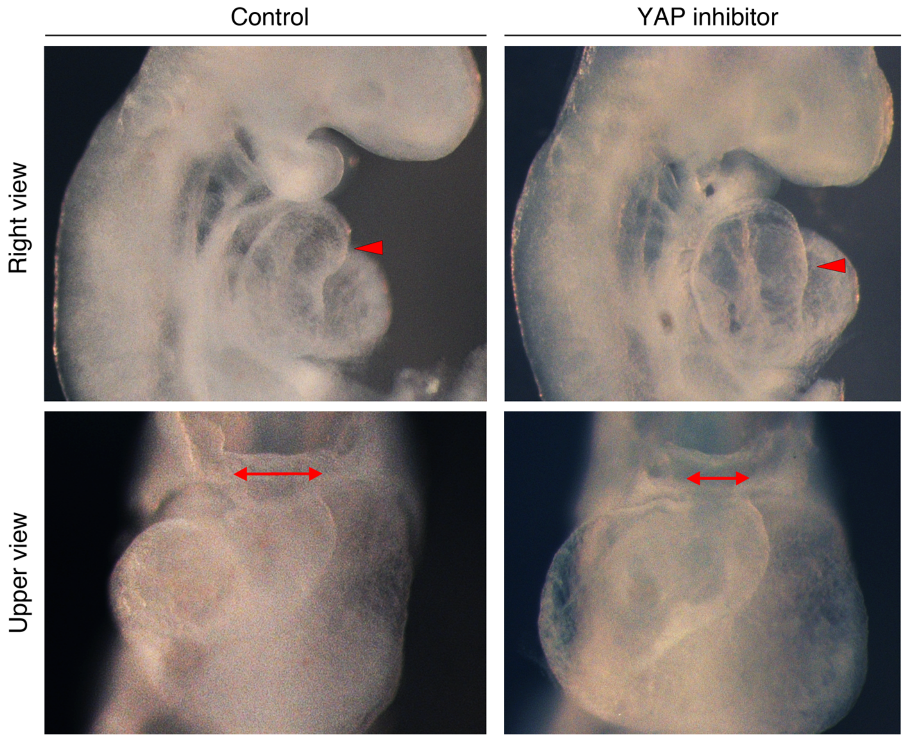

Supplementary Figure 9. YAP inhibition in embryo culture. E9.5 embryos after 24h of culture shows that embryos cultured in the presence of the YAP inhibitor Verteporfin form a shorter and dilated outflow tract with a narrow base of the distal OFT.

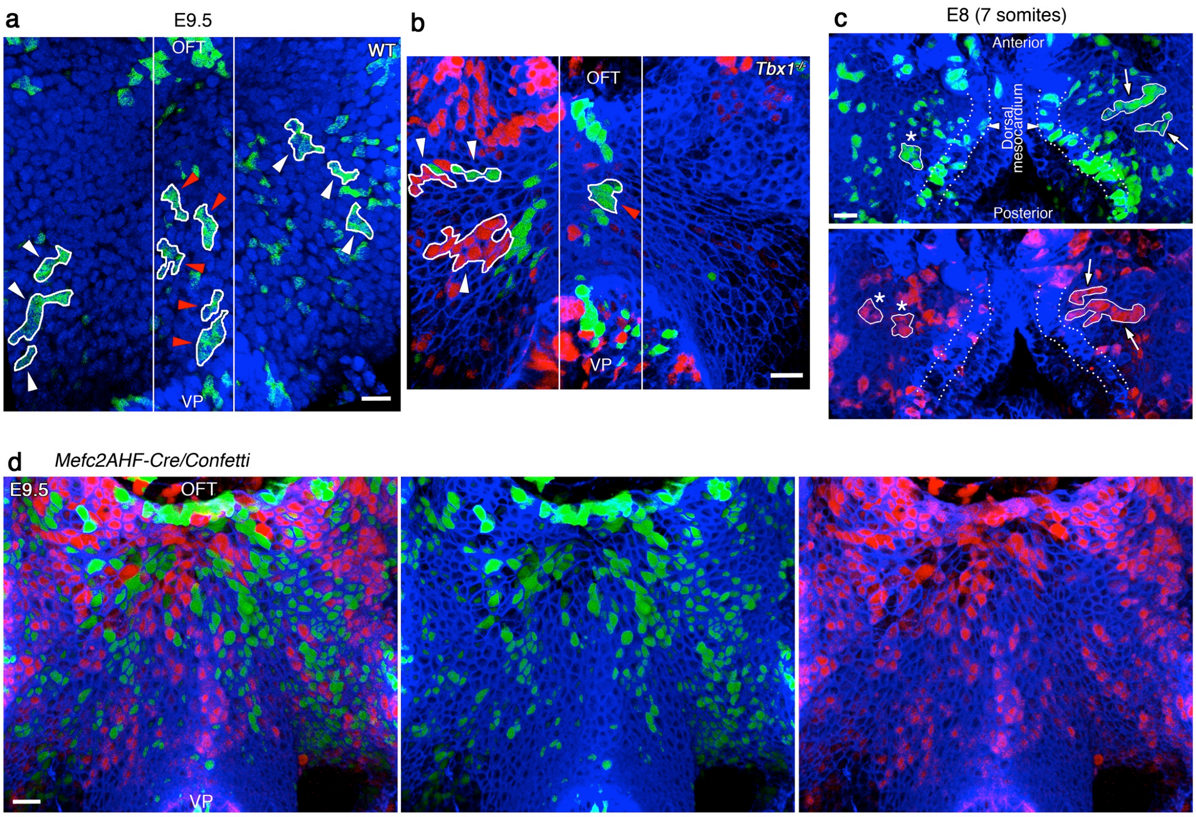

Supplementary Figure 10. ***Mesp1-Cre/Rosa-Confetti*** **and** ***Mef2c-AHF-Cre/Rosa-Confetti*** **labelled clusters in the DPW**. (**a**) E9.5 wildtype embryo showing *Mesp1-Cre;Rosa-Confetti* YFP clusters in the left and right DPW (white arrowheads) elongated on axes directed towards the OFT, and clusters in medial region (red arrowheads) elongated toward the OFT and venous pole. (**b**) E9.5 *Tbx1*$^{-/-}$ embryo showing YFP (green) and RFP (red) clusters. Clusters are less elongated and more oriented on a left-right axis. (**c**) WT E8 embryo (7 somites) showing YFP (green) and RFP (red) clones. Note round isolated cell clusters (white asterisks) and others elongated on the left-right axis (white arrows). (**d**) *Mef2c-AHF-Cre*;*Rosa-Confetti* E9.5 embryo showing YFP and RFP labelled cells in the DPW but few isolated clusters compared to those obtained using *Mesp1-Cre*. OFT: outflow tract, VP: venous pole. Scale bars: 30μm.
